# Single-cell characterization of the gastrointestinal HIV reservoir reveals heterogeneous cellular phenotypes

**DOI:** 10.1172/JCI196536

**Published:** 2025-12-23

**Authors:** Jackson J. Peterson, Shipra Chandel, Katherine James, Elizabeth S. Bennett, Vincent Wu, Cory H. White, Brigitte Allard, Matthew Clohosey, Taylor Whitaker, Caroline Baker, Susan Pedersen, Anne F. Peery, Cynthia L. Gay, Michael R. Betts, David M. Margolis, Nancie M. Archin, Edward P. Browne

**Affiliations:** 1 Department of Microbiology and Immunology and; 2 UNC HIV Cure Center, University of North Carolina at Chapel Hill, Chapel Hill, North Carolina, USA.; 3 Department of Microbiology, University of Pennsylvania Perelman School of Medicine, Philadelphia, Pennsylvania, USA.; 4 Merck, Merck Exploratory Science Center, Cambridge, Massachusetts, USA.; 5 Division of Gastroenterology and Hepatology, Department of Medicine, and; 6 Division of Infectious Diseases, Department of Medicine, University of North Carolina at Chapel Hill, Chapel Hill, North Carolina, USA.

**Keywords:** AIDS/HIV, Immunology, Transcriptomics

## Abstract

Human gastrointestinal (GI) tissues are a major site of HIV-1 viral persistence, but the nature of the GI reservoir remains poorly described. To characterize the GI HIV reservoir, we profiled cells from GI tissue and matched PBMCs from 10 people with HIV on antiretroviral therapy using single-cell RNA sequencing. We identified distinct compartment-specific patterns of gene expression, highlighting key differences between blood and colon CD4^+^ T cell populations. vRNA^+^ cells from both blood and GI tissue were heterogeneous and found in multiple subtypes of CD4^+^ T cells, although vRNA^+^ cells were particularly enriched in cells with Th17 or Treg17 phenotypes. Transcriptomic comparison of HIV vRNA^+^ and vRNA^–^ T cells revealed 116 differentially expressed genes that were associated with HIV infection, including *ZBED2*, *MAF*, and *IL17F*. These data provide what we believe to be new information regarding the GI-resident HIV reservoir and suggest that compartment-specific patterns of gene expression are associated with HIV infection.

## Abstract 2

In this study we use single cell RNA sequencing to characterize HIV infected cells in the blood and colon of people with HIV on therapy.

## Introduction

A reservoir of HIV-infected cells that persists in people with HIV (PWH) despite viral suppression by antiretroviral therapy (ART) is the primary obstacle to an HIV cure (1, 2). Understanding the nature of this reservoir is required to identify HIV cure approaches capable of targeting all reservoirs. However, to date, our understanding of the HIV reservoir has been mostly derived from characterization of virus-infected cells in circulating T lymphocytes found in the peripheral blood and in lymph nodes. By contrast, little is known about viral persistence in tissues such as the gastrointestinal (GI) tract or the cellular phenotype of the infected cells that reside in these tissues. The GI tract is composed of an internally facing mucosal surface, an epithelial layer, and a basal lamina propria (3). Approximately 85% of human lymphocytes are localized to the GI tract (3–5), and immune cells are distributed throughout this tissue, including cells within discrete secondary lymphoid structures, such as Peyer’s patches and lymphocyte populations that reside in the lamina propria and the intraepithelial spaces. Immune cells in the GI tract are heterogeneous and include classical α/β CD4^+^ and CD8^+^ T cells, γ/δ T cells, IgA-expressing plasmablast B cells, innate lymphoid cells, myeloid cells, and mucosal associated invariant T cells (3). Tissue-resident memory cells localize to the epithelium and lamina propria after encountering antigen in the spleen or gut-associated lymphoid tissue (6, 7). Trm cell retention in the GI tract is facilitated by upregulation of the cell-surface proteins CD69 and CD103 and the downregulation of SELL/CD62L, CCR7, and S1PR1 (6, 8–11).

The GI tract is a major site of HIV replication, pathogenesis, and persistence. Extensive viral replication and CD4^+^ T cell depletion occur in the GI tract during the first weeks of acute HIV infection (12–14) or SIV infection (15–18). Systemic inflammation during HIV infection is driven, in part, by localized inflammation in the GI tract, leading to compromised epithelial barrier integrity and microbial translocation (13, 19–21). Even in ART-suppressed PWH, inflammatory signatures persist in the GI tract (14, 20, 22). Similar to the blood, a population of HIV-infected cells persists in the gut mucosa and associated lymphoid tissue of PWH on ART (22, 23), but only a handful of reports have characterized the HIV reservoir in GI tissues of ART-suppressed PWH. CD4^+^ T cells from the GI tract of ART-suppressed PWH have been reported as containing higher levels of total proviral DNA (22, 24–26) than peripheral blood CD4^+^ T cells, and HIV DNA has also been detected in GI-resident macrophages (27–29). However, it is unclear whether higher frequencies of replication-competent or intact provirus are present in the GI tract versus peripheral blood, as measured by assays like the quantitative virus outgrowth assay (30) or the intact proviral DNA assay (31). Studies on the cellular distribution of persistent HIV in the gut have implicated effector memory CD4^+^ T cells as the predominant GI reservoir (25). More recently, it was reported that, for T cells in both the colon and blood, CCR6^+^ memory CD4^+^ T cells constituted a substantial portion of the HIV reservoir, suggesting that CCR6 could potentially be used as a marker to enrich for gut and blood cells containing provirus (23). Identifying the molecular characteristics of the tissue-resident HIV reservoir and how these molecular characteristics impact viral transcription will provide important insight into HIV persistence and transcriptional latency.

In this study, we use a single-cell omics approach to characterize the HIV reservoir in the GI tract of 10 ART-suppressed PWH and to define the relationship of the GI reservoir to the peripheral blood reservoir. We applied single-cell RNA sequencing (scRNAseq) and cellular indexing of transcriptomes and epitopes (CITEseq) to immune cells from both the gut and blood to define the transcriptomic profile of cells in these compartments. Overall, we observed that HIV-infected cells in both blood and colon samples are heterogeneous and can be found in numerous subclusters of CD4^+^ T cells. Nevertheless, we found enrichment of HIV-infected cells in specific subclusters of CD4^+^ T cells, and we observed differences between HIV-infected cells and uninfected cells that may provide some insights into viral persistence in peripheral tissues.

## Results

### Study participants.

10 PWH on ART and 1 HIV-seronegative individual were recruited to donate rectosigmoid biopsies and leukapheresis products or whole blood through the University of North Carolina (UNC) Global HIV Prevention and Treatment Clinical Trials Unit and the UNC Center for AIDS Research HIV Clinical Cohort. The study was approved by the UNC Biomedical Institutional Review Board. Informed consent was obtained from all participants prior to study enrollment. The PWH cohort was 80% male and 20% female; participants had a median age of 59 years (IQR, 43–61 years), had a median CD4 count of 880 cells/μL (IQR, 588–1139 cells/μL), and had a median pre-ART CD4 nadir of 345 cells/μL (IQR, 179.5–389.5 cells/ μL). At the time of sample collection, individuals had been diagnosed with HIV for a median of 16 years (IQR, 13.3–23.2 years), treated with ART for a median of 12.8 years (IQR, 11.2–19.8 years), and durably suppressed (<50 copies/mL) for a median of 11.2 years (IQR, 5.7–15.4 years) (Supplemental Table 1; supplemental material available online with this article; https://doi.org/10.1172/JCI196536DS1).

### Single-cell proteomic and transcriptomic profiling of colon and blood cells from PWH on ART.

**Figure 1:**
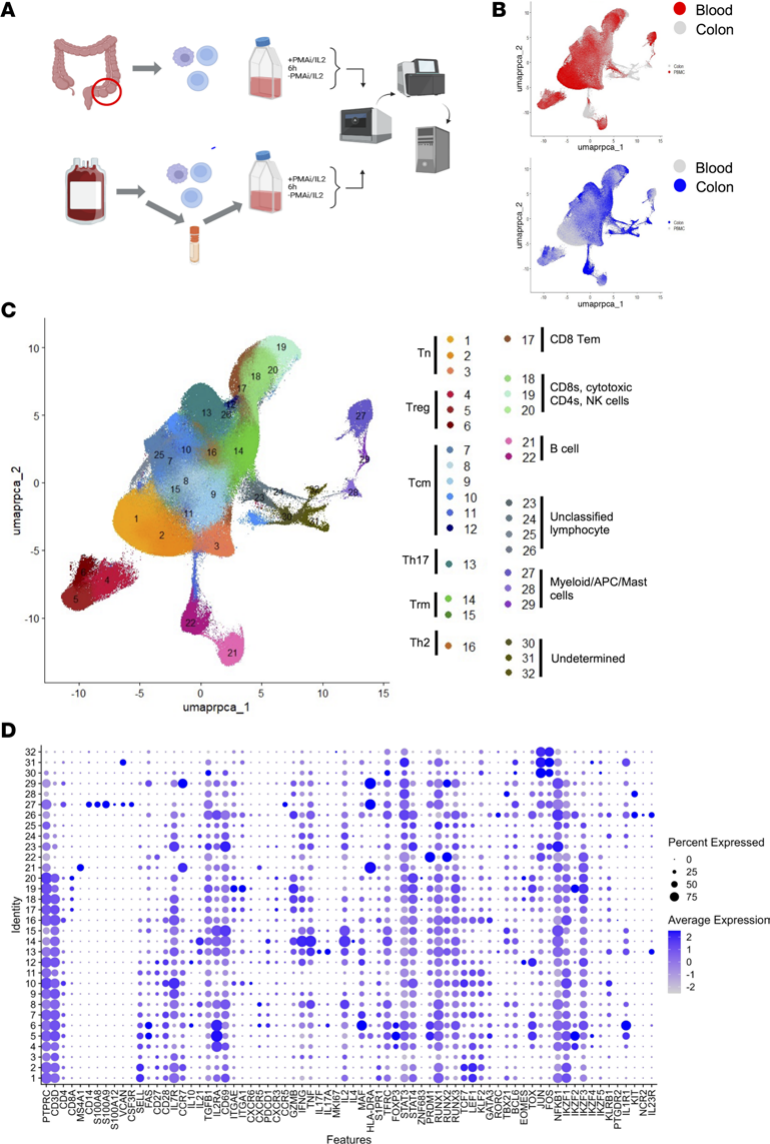
Single-cell proteomic and transcriptomic profiling of colon and blood cells from people with HIV. (**A**) Schematic overview of experimental design and sample processing pipeline. (**B**) Uniform manifold approximation and projection (UMAP) visualization of the combined dataset of scRNAseq and surface protein abundance for matched colon and blood cells from 10 people with HIV (PWH) on antiretroviral therapy (ART) and 1 HIV-seronegative participant. Visualization shown is after reciprocal principal component analysis (RPCA) correction. Top: Blood-derived cells highlighted in red. Bottom: Colon-derived cells highlighted in blue. (**C**) UMAP visualization of cell clusters colored and labeled by annotated cell type. (**D**) Dot plot of gene expression levels from scRNAseq dataset, including both stimulated and unstimulated cells, organized by clusters (*y* axis) and gene of interest (*x* axis). Shown are key markers used in identification and annotation of cell types.

To define features of the HIV-1 reservoir in GI tissue–resident immune cells, fresh rectosigmoid pinch biopsies from the 10 PWH on ART and the HIV-seronegative individual were dissociated to obtain single-cell suspensions. In parallel, we isolated PBMCs from the same individuals. Both PBMCs and GI cells were stimulated for 6 hours with PMA/ionomycin and IL-2, or with control vehicle, before performing scRNAseq/surface protein analysis (Figure 1A). For some samples (donors 4–11), magnetic CD4^+^ T cell^+^ isolation was performed to increase the frequency of CD4^+^ T cells in the cell suspension. For blood-derived samples from 3 donors, we also performed a combined analysis of single-cell transcriptomes and surface protein abundances (CITEseq) using a panel of barcoded antibodies against 135 different surface proteins and 6 isotype controls. The overall scRNAseq/surface protein dataset was assessed using a computational analysis pipeline (Supplemental Figure 1) that included comprehensive quality control (QC) analysis to exclude empty droplets, doublets, or dying cells and the combination of all samples into a single data object. After filtering, we acquired high-quality single-cell transcriptomes from 394,107 cells (161,171 cells from colon and 232,936 cells from peripheral blood) (Supplemental Table 2). Comparison across donors and sample conditions demonstrated similar QC metrics across donors, compartment, and treatment conditions (Supplemental Figure 2, A–C).

We first visualized the scRNAseq dataset from all 11 donors with uniform manifold and projection (UMAP) (Figure 1, B and C). Initial examination of the data revealed heterogeneity driven by the stimulation condition and, to a lesser extent, the tissue compartment (Supplemental Figure 2D). We then employed batch correction with reciprocal principal component analysis (RPCA) to identify corresponding cell types across samples. In the absence of RPCA, unstimulated and PMA/IL-2–stimulated cells were separated on the UMAP plot, indicating the potent effect of this stimulation condition on the transcriptome of both blood and colon cells. Blood and colon cells were also somewhat separated on the UMAP plot, indicating transcriptomic differences between the anatomical compartments. After RPCA, cells from each condition and compartment were aligned across sample conditions, enabling the visualization of shared characteristics in UMAP projections and neighbor finding while still retaining clear compartment-specific cell populations (Figure 1B and Supplemental Figure 2D). Examining the data from the 11 donors separately in the RPCA-corrected UMAP plot, we observed that the data from different donors largely overlapped, suggesting that the overall cluster structure to the data was independent of the donor (Supplemental Figure 3).

We then employed graph-based neighbor finding and clustering to define transcriptionally distinct groups of cells within the data. Across the entire dataset, we identified 32 distinct cellular clusters. We used a manual annotation approach in which annotation was performed by examining expression of a panel of known lineage marker genes (Figure 1D and Supplemental Table 3) and the differentially expressed genes (DEGs) detected in each cluster for unstimulated cells to avoid potential activation-induced masking of cell identity (Supplemental Figure 4). The annotation of cluster identities was further informed by parallel surface protein (CITEseq) analysis from peripheral blood cells (Supplemental Figures 5 and 6) and analysis of cytokines induced by stimulation (Supplemental Figure 7). Of the 32 clusters, 30 were positive (expressed in >25% of cells) for *PTPRC* (CD45) RNA, an immune cell lineage marker (Figure 1D). Furthermore, 20 clusters were robustly positive (expressed in >50% of cells) for RNA encoding the T cell lineage marker CD3D. We renamed the clusters to form numerically close groupings, such that clusters 1–20 contained predominantly CD3^+^ T cells while other immune cell populations form clusters 21–29.

The predominant site of HIV infection and persistence is CD4^+^ T cells, leading us to focus our analysis on CD4^+^ T cells. While *CD4* RNA was overall sparsely detected, *CD4* RNA and CD4 surface protein staining revealed *CD4* expression in all T cell clusters except for cluster 19, which we assessed as likely containing a mixture of NK cells (NCAM1/CD56^+^) and CD8^+^ T cells. *CD8* RNA and CD8 surface protein staining were primarily detected in clusters 17–20; these clusters also expressed transcripts for the cytotoxic effector *GZMB*, even in the absence of stimulation (Supplemental Figure 7). Thus, clusters 17–20 likely consist of a mixture of CD8^+^ T cells, NK cells, and CD4^+^ T cells with a cytotoxic phenotype.

We then identified subsets of functionally distinct cells within the CD4^+^ T cell clusters (clusters 1–16). Clusters 1, 2, and 3 were identified as naive or naive-like T (Tn) cells owing to the absence of the FAS/CD95 surface marker that defines antigen-experienced T cells as well as the presence of the Tn surface protein marker CD45RA (Figure 1D and Supplemental Figure 5). Clusters 1 and 2, and to a lesser extent cluster 3, also expressed comparatively high levels of transcripts for genes encoding surface markers associated with Tn and central memory T (Tcm) cells, including *SELL/CD62L*, *CCR7*, *CD28*, *IL7R/CD127*, and *S1PR1* (32, 33). Additionally, these cells expressed the Tn/Tcm-associated transcription factors (TFs), *TCF7*, *LEF1*, and *KLF2*, that are important for a long-lived quiescent cellular phenotype (34). Clusters 4, 5, and 6 were separated from the main clusters of T cells and were identified as Tregs based on high levels transcripts for the IL2 receptor (*IL2RA*) and the Treg lineage TF *FOXP3* (Figure 1D) (35). Cluster 4 had comparatively lower expression of *FAS/CD95* and may be partially composed of naive Tregs. Cluster 6 exhibited a distinct transcriptional profile, with high levels of expression for *IL1R1* and the TFs *MAF* and *IKZF3*, whereas clusters 4 and 5 exhibited high levels of *IKZF2* expression. Furthermore, unlike the Treg clusters 4 and 5, stimulated cells in cluster 6 expressed *IL10*, *IL17A*, and *IL17F* and may represent a previously described population of intestinal Tregs that expresses both Treg (FOXP3^+^) and Th17 (IL17A^+^, IL17F^+^) characteristics, referred to as Treg17 cells (36, 37). We identified clusters 7–12 as long-lived Tcm cell clusters owing to the presence of *FAS/CD95* RNA indicating antigen-experienced cells; expression of the Tn/Tcm TFs *CCR7*, *LEF1*, *TCF7*, and *IL7R* (Figure 1D); and the lack of surface CD45RA protein expression (Supplemental Figure 5). Cluster 12 expressed *TOX* and *EOMES* suggesting the presence of exhausted T cells, a dysfunctional cell state best characterized in chronic infections and cancer (38). Cluster 13 cells expressed *IL17F* and *IL17A* after stimulation, suggesting the presence of Th17 cells; this designation is supported by expression of *IL23R* and the TF *RORC* (39). Finally, cluster 14 and 15 were annotated as tissue-resident memory CD4^+^ T cells (Trm cells), based on expression of *CD69*, *ITGE*, *S1PR1*, and *KLF2*, while cluster 16 was annotated as containing Th2 cells based on high expression of *GATA3* (40).

### Blood and colon T cells exhibit distinct subpopulation abundances and transcriptomic profiles.

**Figure 2:**
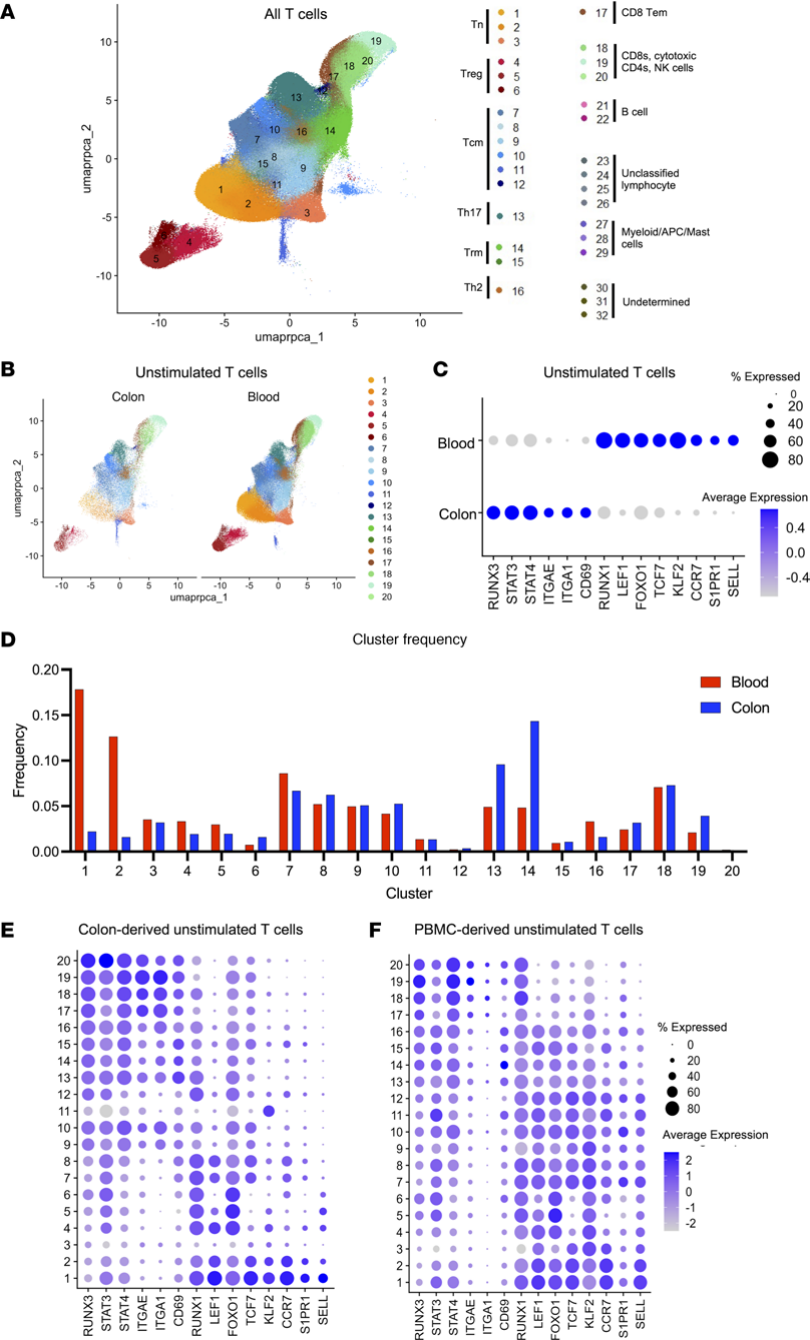
Blood and colon T cells exhibit distinct population abundances and transcriptomic profiles. (**A**) UMAP visualization of combined T cell clusters 1–20. (**B**) UMAP visualization of unstimulated T cells from the blood and colon, with the different compartments shown separately. (**C**) Dot plot comparing expression for selected differentially expressed transcription factor and surface markers between unstimulated blood and colon CD4^+^ T cells. (**D**) Abundance of cells within individual T cell clusters in each compartment, as a fraction of all cells in the compartment. Data from colon cells are shown in blue; data from blood cells are shown in red. (**E** and **F**) Dot plots showing expression of transcription factor and surface markers in unstimulated colon-derived (**E**) and blood-derived (**F**) T cells within each cluster.

We next restricted our analyses to the T cell clusters (clusters 1–20) (Figure 2A) and compared blood T cells to colon T cells. From a visual observation of the UMAP projection, we noted that colon and blood cells exhibited different distributions across the transcriptomic clusters (Figure 2B). To assess global differences between blood and colon T cells, we identified DEGs between cells from these compartments. Among all T cells, 1,311 genes had higher levels of expression in colon T cells, while 2,438 genes had higher levels of expression in blood T cells (P_adj_ < 0.05, log_2_ fold change > 0.3) (Supplemental Figure 8A and Supplemental Table 4). When we examined the top 500 upregulated genes in blood or colon, we observed significant (*P*_adj_ < 0.05) enrichment for several specific biological pathways. In genes that were more highly expressed in colon T cells, the most enriched pathways from the MSigDB database (https://www.gsea-msigdb.org/gsea/msigdb) were “TNF-alpha signaling via NF-kB,” “IL-2/STAT5 Signaling,” and “Inflammatory Response,” while in the genes that were more highly expressed in blood T cells, “IFNa Response” and “IFNg Response” were the most enriched (Supplemental Tables 5 and 6). Notably, when we examined genes that regulate lymph node homing and retention (*CCR7*, *S1PR1*, and *SELL/CD62L*) these genes were all more highly expressed in blood T cells, while 2 integrins that promote binding of cells to extracellular matrix in tissues (*ITGAE* and *ITGA1*) as well as the T cell tissue retention and activation marker (*CD69*) (11) were all expressed more highly in colon-resident T cells (Figure 2C). We also examined several functionally important T cell TFs (41, 42) and observed that *RUNX3*, *STAT3*, and *STAT4* had higher expression in colon-derived T cells, while *RUNX1*, *LEF1*, *FOXO1*, *TCF7*, and *KLF2* were more highly expressed in blood-derived T cells (Figure 2C). When we considered only the unstimulated samples, we also observed several thousand DEGs between colon and blood T cells. Among unstimulated T cells, 2,113 genes were expressed at higher levels in colon T cells and 735 genes were expressed at higher levels in blood T cells (Supplemental Table 7 and Supplemental Figure 8B).

When we examined the proportional abundance of each of the clusters within blood or colon cells, we observed that certain clusters were preferentially represented in one of the compartments (Figure 2D). In particular, we observed that Tn cells were much more abundant in blood, while Treg17, Th17, and Trm cells were more abundant in colon tissue. Thus, colon and blood T cells are different with respect to the subtypes of T cells present. We then examined expression of selected sets of genes across the different clusters within both unstimulated blood and unstimulated colon T cells, including TFs and tissue retention molecules (Figure 2, E and F); known HIV expression–regulating TFs, NF-κB and AP1 (Supplemental Figure 8, C and D); chemokines and chemokine receptors (Supplemental Figure 8, E and F); and interferon-stimulated genes (Supplemental Figure 8, G and H). We observed that the pattern of elevated expression of tissue retention markers (*ITGAE*, *ITGA1*, and *CD69*) was present across most colon T cell clusters, consistent with the higher fraction of cells with a tissue-resident phenotype in the colon (Figure 2, E and F). Similarly, genes that exhibited elevated expression in blood-derived T cells (*LEF1*, *TCF7*, and *KLF2*) had higher across expression across multiple subclusters of blood T cells. Overall, these data identify important differences between the composition and molecular phenotype of cells in the colon and the blood of PWH.

### HIV RNA^+^ cells display heterogeneous phenotypes in the blood and colon of PWH on ART.

**Figure 3:**
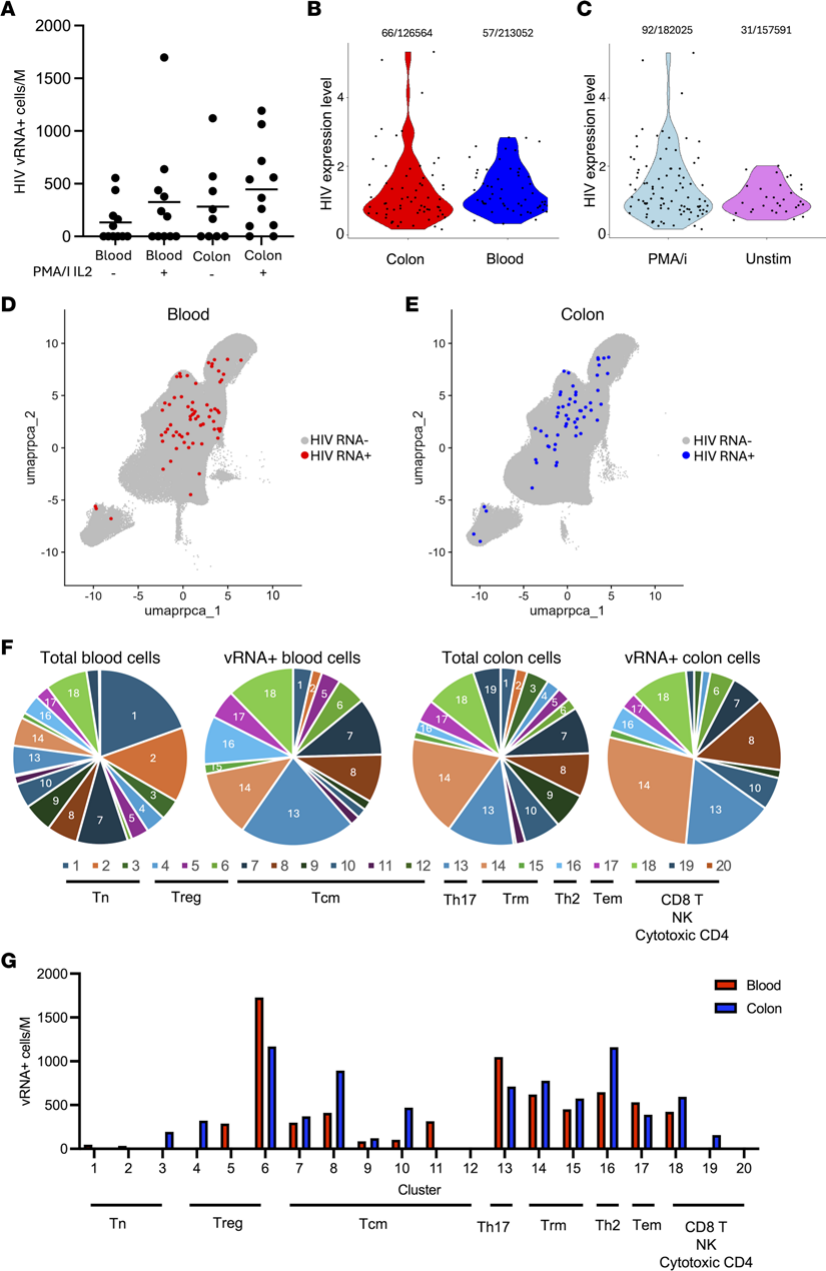
HIV RNA^+^ cells display heterogeneous phenotypes in the blood and colon of PWH. (**A**) The frequency of cells with detectable viral RNA per million cells is displayed for each donor, separated by tissue of origin (blood/colon) and condition (unstimulated/PMAi/IL-2 stimulated). Horizontal bar represents mean values for each column. Each dot represents data from an individual donor. (**B**) Violin plot of normalized HIV expression level for each infected CD4^+^ T cell within the dataset divided by compartment. Blood cells are shown in red; colon cells are shown in blue. Each dot represents a single infected cell. (**C**) As in **B**, but with the data subdivided by stimulation condition. PMAi/IL-2 cells are shown in pale blue, and unstimulated cells are shown in purple. (**D**) UMAP visualization of the combined dataset with vRNA^+^ cells from the blood compartment highlighted in red. (**E**) As in **D**, but with vRNA^+^ cells from the colon compartment highlighted in blue. (**F**) Pie chart showing proportion of vRNA+ cells and total cells in each transcriptomic cluster for blood and colon cells separately. (**G**) Bar chart of the frequency of vRNA^+^ cells within each transcriptomic cluster for blood and colon tissue. Tem, effector memory T cell.

We next examined the expression of HIV RNA transcripts (vRNA) across the cell populations by alignment of the data to a consensus clade B HIV reference genome (43). Although this approach likely underestimates the true abundance of infected cells, due to the limited depth of sampling with scRNAseq and the presence of transcriptionally silent proviruses, this approach nonetheless can be used to characterize a subset of infected cells. Across cells from all 10 PWH, we identified a total of 125 cells with vRNA, 123 of which were from T cell clusters (Supplemental Table 8). The 2 HIV vRNA^+^ cells identified outside of these T cell clusters were found in clusters annotated as “unclassified lymphocytes.” To simplify analyses, we then focused on the 123 HIV vRNA^+^ cells within the 20 T cell clusters. 66 of the HIV vRNA^+^ cells were found within the colon T cells, and 57 were found within the blood T cells. The number of HIV vRNA^+^ cells within each sample was highly variable across the donors, with values ranging from 0 to 21, and the frequency of vRNA^+^ cells per million ranging from 0 per million (/M) to 1,698/M (Figure 3A). As expected, vRNA^+^ cells were more numerous in the PMA/IL-2–stimulated samples than in the unstimulated samples (92 vRNA^+^ cells vs. 31 vRNA^+^ cells respectively). The frequency per million of vRNA^+^ cells in unstimulated colon samples compared with unstimulated blood cells (mean 296/M vs. 143/M) was not significantly different (*P* = 0.41, Mann-Whitney test). Within vRNA^+^ cells, we also examined the expression level of HIV transcripts and found no significant difference in expression for colon cells versus blood cells (*P* = 0.17, Mann-Whitney test) (Figure 3B) or in PMAi/IL-2–stimulated cells versus unstimulated cells (*P* = 0.27, Mann-Whitney test) (Figure 3C).

Next, we examined the distribution of HIV-mapping reads across the viral genome. From the 125 vRNA^+^ cells in the scRNAseq analysis we identified a total of 1,599 HIV-mapping reads derived from 873 unique molecular identifiers (UMIs) (Supplemental Table 9). It should be noted that the raw reads derived from 1 UMI can have multiple start points due to the random fragmentation step after the first PCR of the scRNAseq protocol. We observed several notable features of the distribution of viral reads across the HIV genome. First, many of the reads mapped to the 5′ region of the virus. These included both reads within the 5′ long terminal repeat (LTR), with a large number of reads beginning at the transcriptional start site (TSS), and reads extending past the 5′ LTR into the 5′ region of the Gag open reading frame. Second, additional peaks of viral gene reads were present near nucleotide positions 4,900, 5,400, and 6,000 (Supplemental Figure 9, A and B). Since the library construction protocol used for this study relies on oligo-dT–dependent priming at the poly-A tails of mRNAs and enzymatic fragmentation to generate reads proximal to the poly-A site, this distribution of viral reads distant from the viral poly-A site was initially unexpected. It is unlikely that the TSS proximal reads represent fragments from paused TAR RNAs, since these partial transcripts are not polyadenylated. These observations were consistent across donors, as we observed reads that began at the TSS in 9 donors, reads past the 5′ LTR at the 5′ end of Gag in 7 donors, and peaks near 4.9 kb, 5.4 kb, and 6 kb in 2, 3, and 3 donors respectively. Notably, our findings resemble findings from recent studies using a similar 3′ scRNAseq approach (44, 45). As explored by Schlachetzski et al., the LTR regions are identical in HIV proviruses, so alignments to the 5′ LTR versus the 3′ LTR are difficult to resolve if the read does not extend past the LTR into unique regions (44). However, mapping ambiguity between the 5′ and 3′ LTR does not fully explain the presence of 5′ LTR-mapping reads, since mis-assigned 3′ LTR reads would not extend past the U5 region into Gag. We also identified A-G–rich regions of the HIV genome (13 bp A/G at HXB2 position 778–790, 16 bp A/G at HXB2 position 858–873) internal to the Gag transcript that are likely sufficient to allow internal priming during the reverse transcription step of library construction. Internal priming from A-rich regions is a well-described phenomenon in oligo-dT primed RNA-sequencing libraries (46), and base-pairing between T and G is also thermodynamically stable when G is in an A-rich region (47). Furthermore, the large size of the HIV genome has previously been shown to restrict recovery of full-length viral sequences in near full-length proviral sequencing approaches (48). Thus, we speculate that, in addition to the ambiguity created by identity between the 5′ and 3′ LTRs, internal reverse transcription priming events during library construction may account for a large fraction of viral mapping reads in scRNAseq libraries generated with an oligo-dT based priming approach.

We then examined the distribution of vRNA^+^ cells across the transcriptomic clusters for both blood- and colon-derived T cells (Figure 3, D–G, and Supplemental Figure 10A). Overall, we observed for both blood- and colon-derived cells that, while vRNA^+^ cells were heterogeneous in nature (found in several different transcriptomic clusters) (Figure 3F), they disproportionately originated from a subset of the T cell clusters. In particular, vRNA^+^ cells were predominated by Th17, Trm, Tcm, Th2, and cytotoxic CD4^+^ T cells (Figure 3F) and also differed in relative proportion depending on tissue source. For example, Trm cells made up a larger proportion of the overall pool of infected cells in the colon than in blood cells. While the cluster distribution of vRNA^+^ cells in the blood was quite different from total blood cells, the vRNA^+^ cells in the colon more closely resembled total colon T cells (Figure 3F). It is noteworthy that the population of vRNA^+^ cells in the blood also more closely resembles total colon T cells than total blood T cells with respect to cluster proportions. We thus speculate that a substantial fraction of the vRNA^+^ cells detected in the blood originated from colon tissue.

We then examined the proportional abundance of vRNA^+^ cells as a frequency per million cells for each cluster (Figure 3G and Supplemental Figure 10A). Interestingly, Treg17 cells and Th17 cells exhibited the highest proportional abundance of vRNA^+^ cells, with Tcm and Trm clusters also exhibiting a relatively high abundance of vRNA^+^ cells. Th2 cells in the colon also exhibited a relatively high frequency of HIV RNA–expressing cells. By contrast, vRNA^+^ cells were relatively scarce in cells with a Tn phenotype for both compartments. We also examined the expression level of HIV within the vRNA^+^ cells across the different clusters to determine whether specific cell types are more prone to elevated HIV expression. A broad range of HIV RNA expression was evident across cell clusters, but no statistical difference between the clusters was detected (*P* = 0.197 by Kruskal-Wallis test) (Supplemental Figure 10B), possibly due to the small number of vRNA^+^ cells in some clusters. Additional data may reveal evidence for differential expression of HIV proviruses in different cellular environments.

### The frequency of vRNA^+^ cells in the colon is correlated with the frequency of vRNA^+^ cells in blood.

**Figure 4:**
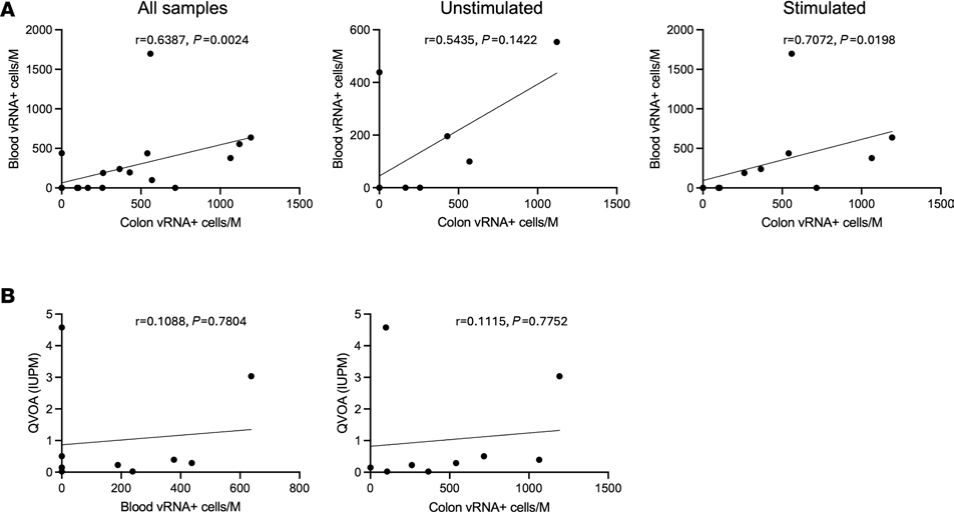
The frequency of vRNA^+^ cells in the colon is correlated with the frequency of vRNA^+^ cells in blood. (**A**) Scatter plot of frequency of vRNA^+^ cells in blood cells vs. vRNA^+^ cells in colon-derived T cells for all conditions (left), unstimulated cells (middle), and stimulated cells (right). Spearman’s correlation coefficients and *P* values are shown. Each data point represents a single participant. (**B**) Scatter plots of the frequency of vRNA^+^ cells in blood cells (left) or colon-derived T cells (right) vs. infectious units per million cells (IUPM) calculated from a quantitative viral outgrowth assay (QVOA) of blood CD4^+^ T cells for each PWH. Spearman’s correlation coefficients and *P* values are shown. Each data point represents a single participant.

To better understand the relationship between the blood reservoir and the colon reservoir, we next assessed the level of correlation between the frequency of HIV vRNA^+^ cells in these compartments. When we examined the correlation between blood and colon vRNA^+^ cells for all samples, both unstimulated and stimulated, we observed a significant correlation across the cohort (*r* = 0.6387, *P* = 0.0024 Spearman’s correlation) (Figure 4A). When we separated the two conditions (stimulated and unstimulated), we still observed a significant correlation between vRNA^+^ cells in blood and colon for stimulated samples (*r* = 0.7072, *P* = 0.0198) but not for unstimulated samples (*r* = 0.5435, *P* = 0.1442). Since a subset of the study participants had previously had their replication-competent blood reservoir quantified by quantitative viral outgrowth assay (QVOA), we also examined the correlation between infectious units per million cells (IUPM) measured by this assay and the frequency of vRNA^+^ cells. Notably, the frequency of vRNA^+^ cells in neither the blood nor the colon correlated with the IUPM measured by QVOA (Figure 4B). Thus, while the frequency of inducible vRNA^+^CD4^+^ T cells in the colon correlates with the frequency of inducible vRNA^+^ cells in the blood, we do not observe a clear correlation between the frequency of vRNA^+^ cells and the inducible replication-competent HIV blood reservoir measured by QVOA.

### Identification of DEGs in vRNA^+^ cells.

**Figure 5:**
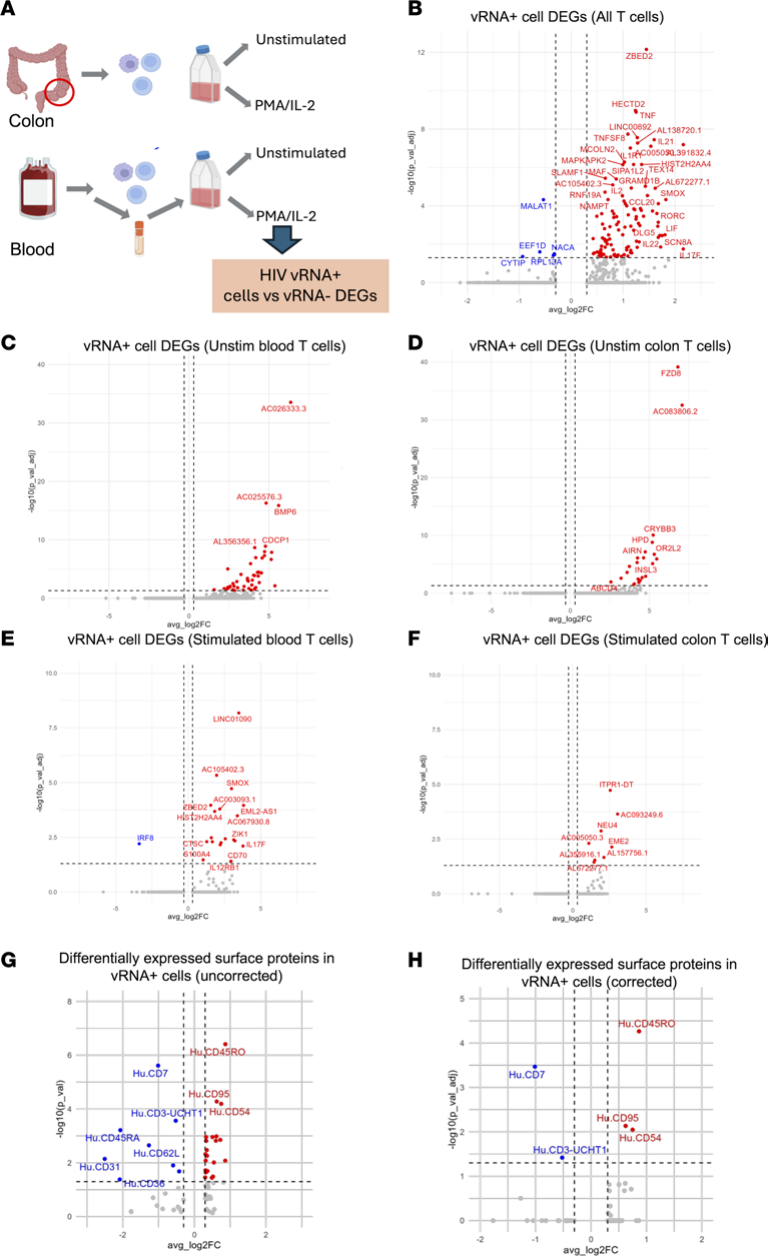
Identification of differentially expressed genes in vRNA^+^ cells. (**A**) Schematic overview showing experimental design. (**B**) Volcano plot of differentially expressed (log_2_ fold change > 0.3, *P*_adj_ < 0.05, Wilcoxon’s rank-sum test) genes in HIV vRNA^+^ cells compared with vRNA^–^ CD4^+^ T cells across all samples (blood and colon, stimulated and unstimulated cells). Upregulated genes in red, downregulated genes in blue. (**C**–**F**) Volcano plots of differentially expressed genes, as in **B**, specifically within the indicated sample conditions. (**G**) Differentially expressed surface proteins between vRNA^+^ cells and vRNA^–^ cells before correction for multiple testing. (**H**) As in **G**, but after Bonferroni’s *P* value correction for multiple testing.

We next examined whether the HIV vRNA^+^ cells exhibit any unique gene expression patterns that could distinguished them from uninfected cells (Figure 5A). First, we compared all HIV vRNA^+^ T cells across both compartments and both stimulation conditions to vRNA^–^ T cells. We identified 111 genes that were expressed to a higher level in vRNA^+^ cells (Figure 5B and Supplemental Table 10). This list included several cytokines (*IL2*, *IL17F*, *IL21*, *IL22*, *IFNG*, *TNF*, and *CCL20*) as well as *ZBED2*, a TF that regulates IFN responses (49); spermine oxidase (*SMOX*), an enzyme that has been proposed to mediate Tat-dependent neurotoxicity (50); and the TF *MAF*. By contrast, only 5 genes, *MALAT1*, *EEF1D*, *NACA1*, *RPL13A*, and *CYTIP*, were transcriptionally downregulated in the HIV vRNA^+^ cell population relative to vRNA^–^ cells. We next compared gene expression between vRNA^+^ and vRNA^–^ T cells within blood or colon, dichotomized by stimulation condition, and observed that 50 genes (including HIV) exhibited elevated expression in vRNA^+^ unstimulated blood T cells compared with vRNA^–^ cells (Figure 5C and Supplemental Table 11), while 23 genes were elevated in unstimulated vRNA^+^ colon T cells (Figure 5D and Supplemental Table 12). Notably, these 2 sets of genes exhibited no overlap, suggesting that DEGs that characterize vRNA^+^ cells in the blood are distinct from the DEGs that define vRNA^+^ cells in the colon. When we looked within the stimulated blood cell dataset, we observed 22 upregulated genes and no downregulated genes in vRNA^+^ cells (Figure 5E and Supplemental Table 13). In vRNA^+^ cells, *ZBED2*, the zinc finger transcriptional repressor *ZIK1*, and *SMOX* were upregulated, along with interferon regulatory factor 8 (*IRF8*). Consistent with our observed enrichment of vRNA^+^ cells within the Th17 compartment, the cytokine *IL17F* also exhibited upregulated expression in vRNA^+^ cells within stimulated blood samples. Within stimulated colon T cells, only 9 genes exhibited differential expression, all of which were higher in vRNA^+^ cells (Figure 5F and Supplemental Table 14). The most significantly upregulated (*P*_adj_ < 0.05) gene in stimulated colon cells was the long noncoding RNA ITPR1-DT, followed by an antisense transcript for *SRCAP*. Some genes associated with Th17 cell identity such as *RORC* and *IL21* also trended high in stimulated vRNA^+^ colon T cells, but these differences were not statistically significant (*P*_adj_ > 0.05). For the blood cell dataset, we also examined differential expression of surface proteins between vRNA^+^ and vRNA^–^ cells. Prior to correction for multiple comparisons, 29 proteins were differentially abundant between vRNA^+^ cells and vRNA^–^ cells across the entire dataset, with 21 proteins upregulated and 8 downregulated (Figure 5G and Supplemental Table 15). Following multiple comparison correction, 5 proteins were found to be significantly differentially abundant — 3 that were higher in vRNA^+^ cells (CD45RO, CD95, and CD54) and 2 that were higher in vRNA^–^ cells (CD3 and CD7) (Figure 5H). These data are consistent with enrichment of vRNA^+^ cells in memory T cells.

To gain further biological insight into the nature of the transcripts that were enriched in HIV vRNA^+^ cells, we analyzed the sets of DEGs within 2 curated databases of gene sets — Kyoto Encyclopedia of Genes and Genomes (KEGG; https://www.genome.jp/kegg/) and MSigDB Hallmark using Enrichr Pathway analysis (51). When we examined the set of 111 genes that exhibited enriched expression in vRNA^+^ cells across all the samples and conditions, we observed that 5 categories from the MSigDB Hallmark sets (52) were significantly (*P*_adj_ < 0.05) enriched: TNF-alpha Signaling via NF-κB, Inflammatory Response, Allograft Rejection, IL-2/STAT5 Signaling, and mTORC1 Signaling (Supplemental Table 16). Within the KEGG gene sets, 19 sets were enriched in these DEGs, including inflammatory bowel disease, Th17 cell differentiation, and NF-κB signaling pathway (Supplemental Table 17). These enrichment patterns suggest that HIV expression within the overall reservoir is associated with specific biological pathways, particularly inflammatory pathways that regulate NF-κB activity and with Th17 differentiation. We also repeated this analysis within single compartments and conditions. For the DEGs associated with vRNA^+^ cells in both unstimulated blood and colon T cells we observed no specific pathway enrichment. For DEGs in vRNA^+^ cells within stimulated blood samples, 3 categories from the KEGG sets were identified as being significantly enriched — inflammatory bowel disease, cytokine-cytokine receptor interaction, and Th17 differentiation (Supplemental Table 18), while no pathway enrichment was observed for HIV vRNA^+^ cell DEGs from stimulated colon T cells, likely due to the small number of DEGs. Overall, these analyses indicate that, despite their heterogeneity, HIV vRNA^+^ cells in both the blood and colon exhibit some differences from vRNA^–^ cells. In particular, blood vRNA^+^ T cells are characterized by elevated expression of genes associated with a Th17 phenotype and with inflammatory bowel disease. We also conclude the small number of DEGs that define vRNA^+^ cells are not conserved between infected T cells in the blood and colon.

## Discussion

Despite the importance of tissue T cells in HIV infection, few studies have investigated the nature of the HIV reservoir in tissues, including the GI tract, and additional biological investigation of the tissue-resident reservoir in PWH is urgently needed. In this study we analyzed single-cell transcriptomes from colon mucosa pinch biopsies and matched peripheral blood samples from a cohort of PWH. We used these data to identify differences between the CD4^+^ T cells of peripheral blood and the GI tract as well as to identify a set of genes that are differentially expressed in HIV RNA–expressing cells in the blood and in the colon. Consistent with previous observations, we observed that the majority of colon-resident T cells exhibit an effector memory phenotype (CD45RA^–^/CD45RO^+^/CD62L^–^), while in the blood, T cells with a naive phenotype (CD45RA^+^/CD45RO^–^/CD62L^+^) are more abundant. Our study also characterized 123 HIV vRNA–expressing cells across the blood and colon T cell samples, allowing us to examine the characteristics of these cells and to compare the HIV-infected cells in blood and colon. Notably, we did not observe any infected cells with a myeloid phenotype. Recent studies have shown that HIV DNA can be found in intestinal macrophages in PWH on ART, although the frequency is much lower than for T cells (29). It is possible that a dissociation protocol that has been optimized for tissue macrophages could recover vRNA^+^ cells within this lineage.

Overall, we observed that the vRNA^+^ cells in both the blood and the colon are heterogeneous and found in several phenotypic clusters of T cells, including in Th17 cells, Tregs, Tn cells, Th2 cells, and others. Nevertheless, for blood cells, we observed that the cluster distribution of vRNA^+^ T cells was distinct from the overall blood T cell population, with Tn cells being underrepresented and cells with a Th17 and Trm phenotype being overrepresented. By contrast, the cluster distribution of vRNA^+^ cells in the colon more closely resembled the overall distribution of colon T cells. It is particularly noteworthy that the vRNA^+^ cell population in the blood resembles total colon T cells more than total blood T cells with respect to overall cluster distribution. This observation, along with the observation that the frequencies of vRNA^+^ cells in blood and colon are correlated, make it tempting to speculate that vRNA^+^ cells that are detected in the blood may have originated in the colon or in other tissues. Nevertheless, vRNA^+^ cells in the colon still exhibited some differential gene expression compared with colon T cells indicating that, even within the colon, HIV may have a preference for specific subtypes of T cells or that virus infection itself can impact the transcriptomic phenotype of the cells. Additional sampling of a greater number of donors and larger populations of cells may be required to definitively validate this hypothesis.

It is particularly intriguing that the cluster with the highest frequency of vRNA^+^ cells in the blood are cells with a Treg17 phenotype, a recently described population of Tregs that is more abundant in the colon (36). Treg17 cells express characteristics of both Tregs (FOXP3^+^) and Th17 cells (IL17^+^), and their function has not been extensively investigated. To our knowledge, no previous studies have highlighted Treg17 cells as a target for HIV infection. Interestingly, genetic studies in mice have shown that the TF MAF, which our data identify as being preferentially expressed in vRNA^+^ cells, plays a key role in the development of the Treg17 lineage, and *Maf*-deficient mice exhibit enhanced intestinal inflammation (37). Additionally, MAF has been shown to bind the HIV LTR and to promote HIV expression (53). Further studies will be needed to clarify the role of MAF-expressing Treg17 cells in HIV infection.

When we compared the transcriptomes of the vRNA^+^ cells to the vRNA^–^ cells across all compartments and conditions, we identified a signature of 116 differentially expressed cellular transcripts. Notably, the 111 upregulated genes in vRNA^+^ cells were enriched for inflammatory pathways and Th17 differentiation, consistent with our observation that vRNA^+^ cells are enriched within the Th17 cluster. These genes that are upregulated in vRNA^+^ cells could potentially be further examined for mechanistic roles in HIV infection or in reservoir persistence. We also observed 5 transcripts that were downregulated in vRNA^+^ cells (*MALAT1*, *EEF1D*, *NACA1*, *RPL13A*, and *CYTIP*). Interestingly, *MALAT1* RNA has been previously identified as a positive regulator of HIV transcription and has been shown to counteract transcriptional repression of HIV by the polycomb-repressive complex 2 (PRC2) (54), and our lab has also recently shown that the T cell TF ETS1 represses HIV expression in latently infected cells by repressing *MALAT1* expression (55). It is possible that establishment of the latent reservoir occurs preferentially in cells with lower *MALAT1* expression. We also examined differential expression of surface proteins in vRNA^+^ cells for blood-derived cells and identified 5 differentially expressed proteins — CD3 and CD7 were lower in vRNA^+^ cells, while CD95, CD54, and CD45RO were elevated in vRNA^+^ cells. These data are consistent with the hypothesis that the transcriptionally active reservoir is enriched in cells with a memory (CD45RO^+^) phenotype. Interestingly, CD7^–^ T cells have previously been shown to represent a subset with low permissiveness for HIV replication that is preferentially expanded during HIV infection (56). Further work will be needed to examine the connection between CD7 expression and the transcriptionally active reservoir.

Over the past few years, several laboratories have used single-cell methods to profile cells from PWH and identify the transcriptomic, proteomic, and epigenomic characteristics of the HIV reservoir (57–59). These datasets have also pointed to a heterogeneous reservoir found in several CD4^+^ T cell subsets, with few differences between the infected cells and uninfected cells being consistently observed across studies. Nevertheless, the number of infected cells that have been profiled through these approaches remains fairly small, and additional biological patterns that are associated with the latent reservoir may emerge as larger scale datasets are generated. Furthermore, the HIV reservoirs found in many solid tissues remain poorly described, and detailed characterization of HIV-infected cells in various tissue contexts will be essential to understanding the overall phenomenon of HIV persistence during ART. Some recent studies have begun to use single-cell profiling approaches to characterize tissue HIV reservoirs (44, 60). Wei et al. used single-cell DOGMA-seq and TREK-seq to profile the transcriptome, surface proteome, chromatin accessibility, and TCR sequence of T cells from PWH on ART to identify 99 HIV-infected cells within gut biopsies (60). Similar to our findings, this study reported that colon CD4^+^ T cells exhibit elevated expression of markers of tissue-resident memory cells, such as *ITGA1* and *ITGAE*. This study also reported that vRNA^+^ cells were enriched within cells with elevated expression of Th17 cells, such as *RORC* and *IL17A*, consistent with our findings. In contrast with this study, our data do not indicate that vRNA^+^ cells in the blood or colon exhibited elevated expression of *BACH2* compared with vRNA^–^ cells from the same compartment. Since both this study and ours rely on relatively limited sampling of gut tissue and vRNA^+^ cells, it is likely that larger studies are needed to fully resolve the phenotype of HIV-infected cells in tissues.

An important limitation of our approach is that we were unable to derive complete information about the viral sequences present in each infected cell from our dataset, owing to the small fragment size and short read length for the scRNAseq platform. Since the reservoir consists of a heterogeneous mixture of intact and defective proviruses (61), it will be important to determine whether cells that contain intact or defective proviruses exhibit different characteristics. Methodologies that allow complete or near-complete viral sequences to be derived in parallel with transcriptomic information will likely provide additional insights into the characteristics of the intact and defective reservoirs. The specific viral transcripts that we have detected by the scRNAseq approach are also largely ambiguous due to the overlapping nature of many HIV transcripts, and it will be interesting to examine whether colon-resident-infected cells exhibit differential splicing of HIV vRNA compared with blood cells. Another limitation of our study is that the biopsy samples used for this study were obtained exclusively from the rectosigmoid location in the colon, and these samples may not represent the HIV reservoir across different locations of the GI tract (62). Developing approaches to obtain biopsy samples from different locations could reveal new characteristics of GI tissue reservoir cells. An additional caveat of our approach is that detection of infected cells relies on the presence of viral RNA reads within the scRNAseq data. Prior work has indicated that only a minor fraction of the HIV reservoir is transcriptionally active at any given point in time (63), and our method thus likely fails to capture a larger population of cells with transcriptionally inactive proviruses. As such, conclusions regarding the overall reservoir composition for both blood and colon compartments should be interpreted with caution. Other approaches, such as using scATACseq to identify cells with accessible HIV DNA, are also biased toward transcriptionally active proviruses, leaving a large fraction of the quiescent reservoir uncharacterized. It is also important to consider that the small sample size of this study (*N* = 10 PWH) and the limited number of HIV-infected cells (125 cells) that were detected could lead to sampling bias influencing the findings. Indeed the pool of vRNA^+^ cells was disproportionately composed of cells from a subset of donors (Supplemental Table 9). As mentioned above, a larger and more comprehensive study is needed to further validate many of these findings.

Nevertheless, our study is one of the first to derive a transcriptomic characterization of HIV-infected cells from GI tract of PWH and provides characterization of tissue-resident infected cells that differ from uninfected cells and from cells in the peripheral blood. In particular, we found that the transcriptionally active reservoir is enriched in specific subtypes of T cells, which could potentially be targeted as part of an HIV cure strategy. Our approach could also serve as a template for much needed additional studies regarding the GI-resident HIV reservoir. Additional data to characterize the spatial environment of the HIV reservoir within tissues will also likely yield valuable insights.

## Methods

### Sex as a biological variable.

Our study cohort consisted of 8 male PWH)and 2 female PWH. Sex was not considered as a biological variable owing to the small cohort size.

### GI tissue dissociation and cell preparation.

After 2 saline enemas, a sigmoidoscopy was performed by a gastroenterologist in an endoscopy suite at UNC Chapel Hill. The exam was performed without sedation and was completed in less than 10 minutes. The endoscope was advanced to the rectosigmoid where 15 single-pass biopsies were taken using standard disposable fenestrated colonoscopy biopsy forceps with a central spike. Samples were delivered in tissue culture media on ice within 2 hours of acquisition. 8–10 pinch biopsies from each individual were dissociated into single-cell suspensions with a CLSPA-based method. To enhance cell recovery, falcon tubes (Corning) and cell-culture dishes were coated with a 1% bovine serum albumin solution for 30 minutes, emptied, allowed to dry for 30 minutes with the lids partially open, and subsequently ultraviolet light–irradiated for 45 minutes. Cell suspensions were then subjected to dead-cell removal (StemCell Technologies) and, for some samples, CD4^+^ isolation (StemCell Technologies). Cells were rested overnight at >1 M/mL in complete RPMI-1640 (Gibco) containing 10 U/mL of IL-2, 10 μM ZVAD-FMK caspase inhibitors (Selleck Chem), and 500 nM Raltegravir (Selleck Chem). Following overnight rest, part of the cell population was treated with PMA/ionomycin (16 nM/1 μM) and IL-2 (100 U/mL) for 6 hours and collected for scRNAseq analysis.

PBMCs were collected via leukapheresis or a large blood draw within 1 month of the GI biopsy collections. For 3 participants, PBMCs were collected within 6 months to 1.5 years of GI biopsy. PBMCs were isolated by Ficoll (Sigma-Aldrich) gradient centrifugation and then viably frozen. PBMCs were analyzed after thawing and resting in complete RPMI-1640 with 10 U/mL IL-2, 10 μM ZVAD-FMK, and 500 nM raltegravir overnight, followed by further resting or stimulation with PMA/ionomycin (16 nM/1 μM) and IL-2 (100 U/mL) for 6 hours as above. Cultures were then subjected to dead cell removal and scRNAseq using a Chromium controller (10xGenomics). All samples exhibited greater than 70% cell viability at the time of sample loading, as measured by Trypan blue exclusion. CD4^+^ T cell magnetic isolation was performed for 8 donors, while samples from an additional 3 donors were processed with unfractionated populations.

### CITEseq surface protein staining.

PBMCs from 3 donors were thawed and rested overnight, as described above. The next day, CD4 cell magnetic isolation was performed on samples from all 3 donors while separate PBMC cultures were retained. Following the 6-hour culture period, cells were washed in PBS and prepared for CITEseq analysis. The TotalSeq-B Universal Human antibody panel (Biolegend, catalog 399904) of lyophilized DNA-tagged antibodies was resuspended and spun down according to manufacturer’s instructions. Meanwhile, cells were treated with Human TruStain FcX (Biolegend, catalog 422301) to prevent antibody binding by human Fc receptors. Next, cells from each donor were stained with Totalseq-B hash-tag oligonucleotide antibodies (Biolegend, catalog 155831, 155833, and 155835) and then added to 3 pools comprising untreated PBMCs, untreated CD4 cells, and PMA/ionomycin-stimulated CD4^+^ T cells. Pooled samples were stained in parallel with the TotalSeq-B Universal Human antibody panel, washed, and prepared for scRNAseq library construction.

### scRNAseq library preparation.

scRNAseq libraries were prepared according to the manufacturer’s instructions (10x Genomics scRNAseq v3.1 3′ sequencing, catalog 1000269). Gel-bead and cell emulsions (GEMs) were generated using the Chromium controller, subjected to reverse transcription, and first-strand cDNA was extracted from GEMs. Purified cDNA was PCR preamplified (12 cycles, 1-minute extension) with primers binding the P7 region and the Template Switching Oligo, cleaned up by solid-phase reversible immobilization (SPRI) beads, analyzed by HS DNA 5000 capillary electrophoresis (Agilent), and concentration calculated by Qubit (ThermoFisher). From this material, 25% was used for Illumina NGS library construction involving fragmentation, end-repair/A-tailing, adapter TA ligation, and SPRI double-sided size selection to remove adapters and unfragmented products. Libraries were amplified with Illumina indexing/sequencing i5 and i7 primers (13 cycles) and then cleaned with SPRI beads before being quantified by Qubit, and the fragment size was determined using a Tapestation (Agilent). Libraries were then pooled at an equimolar concentration and sequencing was performed using NovaSeq or NovaseqX sequencing (Illumina) using the following read format: R1 28bp, i7 10bp, i5 10bp, R2 91bp.

### Data analysis.

Alignment and cell calling was performed on the UNC high-performance computing cluster with cellranger v7.1.0 (10xGenomics) against a custom human HIV reference genome generated from concatenating GRCh38 to an extra chromosome containing consensus clade B HIV sequence fasta and.gtf files and compiling with the cellranger mkref function (43). Alignment was performed for all files using cellranger count function with default parameters but --jobmode=slurm to run on the UNC high-performance computing cluster. Count matrices were imported into Seurat (version 5.0) using custom R code to first import the data matrices and add metadata fields indicating features such as sample of origin, donor, and whether the cells had been treated with PMA/ionomycin or not. Custom R code was further employed to perform QC for high-quality transcriptomes (nFeature_RNA>500, percent.mt<15, and nCount_RNA<100000), remove doublets with scDblFinder, generate summary statistics before and after QC, and add the sample to a combined data matrix. CITEseq gene and feature count matrices were imported with separate R code that performed additional processing of the CITEseq feature counts data, including separating the hash-tag oligo data matrix with the HTODemux command in Seurat. Metadata was then applied to demuxed CITEseq data objects as above. Subgroupings of cells were created using the subset() command in R, which can create a smaller subset of a dataset based on defined features.

### Statistics.

Differential expression tests were performed with the Wilcoxon’s rank-sum test (Seurat FindMarkers) with a minimum log_2_ fold change of 0.3 (log fold change threshold = 0.3), and the analyses were restricted to features that occur in 10% of cells (minimum percentage = 0.1). Correlation analyses were perform using Spearman’s correlation and analyzed using Prism. *P* values of less than 0.05 were considered significant.

### Study approval.

Our cohort consisted of 10 individuals with HIV on suppressive ART and 1 HIV-seronegative participant. Sample collection was performed under supervision of the UNC Chapel Hill Institutional Review Board, under study no.19-2480 which was approved by UNC Biomedical Institutional Review Board. All participants provided written informed consent for the study.

### Data availability.

All underlying data are available via the UNC Dataverse (https://dataverse.unc.edu/dataverse/Brownelab and https://doi.org/10.15139/S3/XYUNVK). Raw sequencing data files are available at GEO (GSE313894). Supporting data values for figures are available in the Supporting Data Values file.

## Author contributions

EPB, NMA, and JJP designed the study. JJP, SC, ESB, KJ, BA, and MC carried out experiments. CB, SP, AFP, CLG and TW provided resources. JJP, CHW, VW, MRB, and EPB performed analysis. EPB, NMA, JJP, DMM, and MRB wrote the manuscript. EPB, NMA, and DMM acquired funding.

## Funding support

This work is the result of NIH funding, in whole or in part, and is subject to the NIH Public Access Policy. Through acceptance of this federal funding, the NIH has been given a right to make the work publicly available in PubMed Central.

- NIH National Institute of Allergy and Infectious Diseases (NIAID 5-R01AI143381), National Institute of Diabetes and Digestive and Kidney Diseases (NIDDK 1R01DK131526), and NIAID (5-UM1AI164567).
- UNC Center for AIDS Research (P30AI050410).
- NIAID UM1 AI164570 BEAT HIV Martin Delaney Collaboratory to MRB and VW.

## Supplementary Material

Supplemental data

Supplemental table 1

Supplemental table 10

Supplemental table 11

Supplemental table 12

Supplemental table 13

Supplemental table 14

Supplemental table 15

Supplemental table 16

Supplemental table 17

Supplemental table 18

Supplemental table 2

Supplemental table 3

Supplemental table 4

Supplemental table 5

Supplemental table 6

Supplemental table 7

Supplemental table 8

Supplemental table 9

Supporting data values

## Version 1 12/23/2025

In-Press Preview

## Version 2 02/16/2026

Electronic publication
