# Supplementary material for: Single-cell characterization of the gastrointestinal HIV reservoir reveals heterogeneous cellular phenotypes
Source: J Clin Invest. 2025 Dec 23;136(4):e196536. doi: 10.1172/JCI196536 (PMC12904708; doi:10.1172/JCI196536)
Supplement: Supplemental data [file jci-136-196536-s098.pdf]

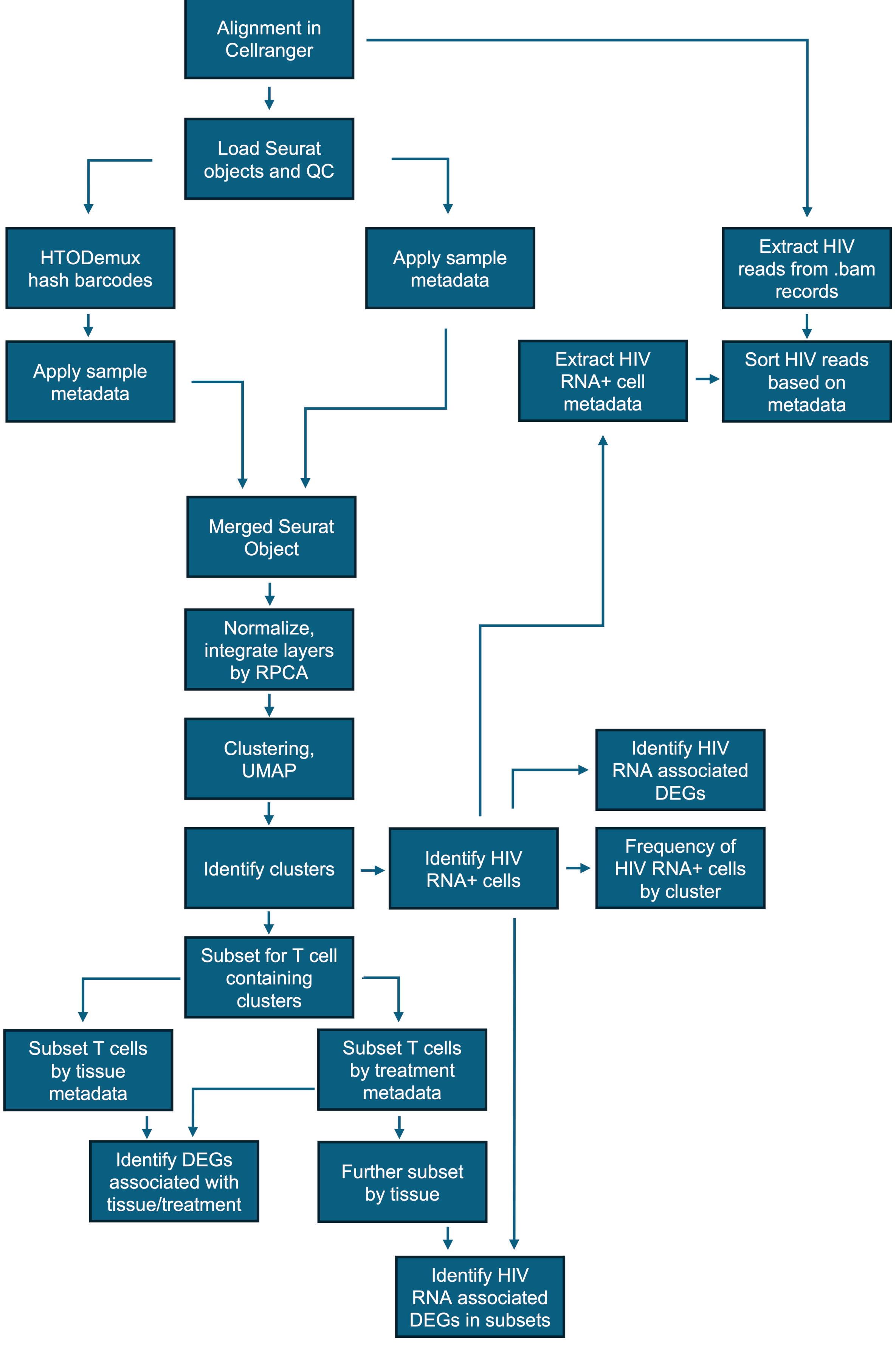
Alignment in Cellranger
Load Seurat objects and QC
HTODemux hash barcodes
Apply sample metadata
Apply sample metadata
Extract HIV reads from .bam records
Extract HIV RNA+ cell metadata
Sort HIV reads based on metadata
Merged Seurat Object
Normalize, integrate layers by RPCA
Clustering, UMAP
Identify HIV RNA associated DEGs
Identify clusters
Identify HIV RNA+ cells
Frequency of HIV RNA+ cells by cluster
Subset for T cell containing clusters
Subset T cells by tissue metadata
Subset T cells by treatment metadata
Identify DEGs associated with tissue/treatment
Further subset by tissue
Identify HIV RNA associated DEGs in subsets

A.

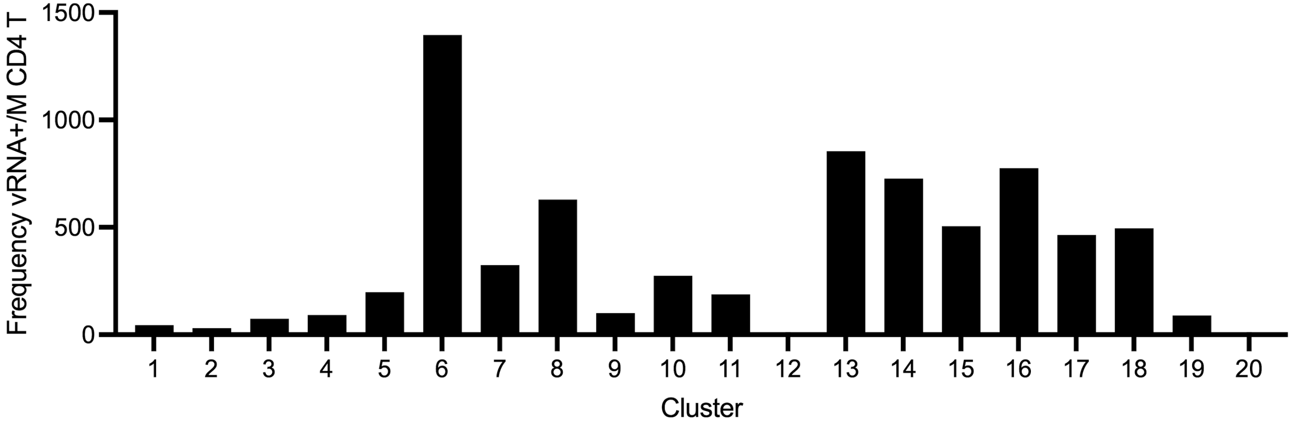

Frequency vRNA+/M CD4 T
1500
1000
500
0
1
2
3
4
5
6
7
8
9
10
11
12
13
14
15
16
17
18
19
20
Cluster

B.

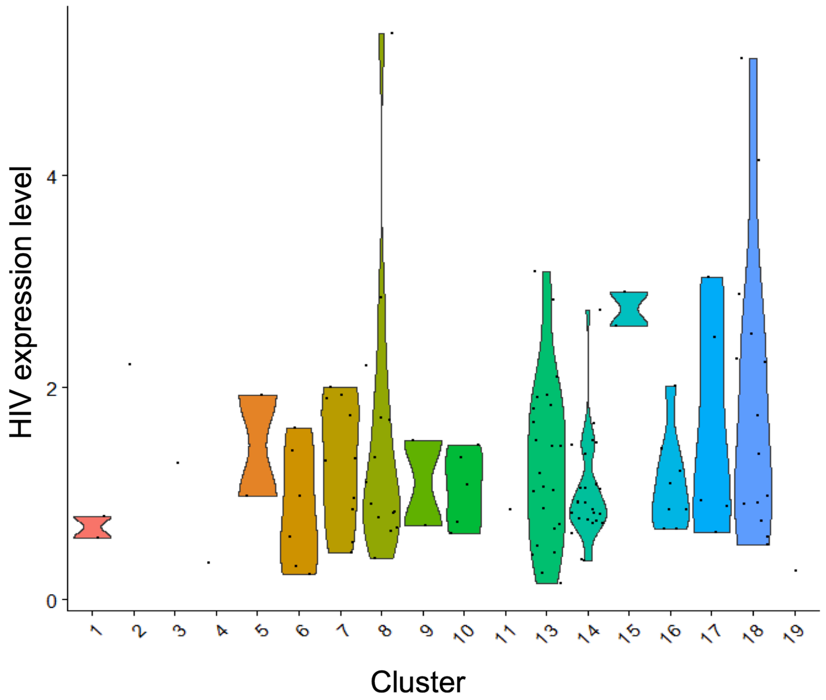

HIV expression level
4
2
0
1
2
3
4
5
6
7
8
9
10
11
13
14
15
16
17
18
19
Cluster

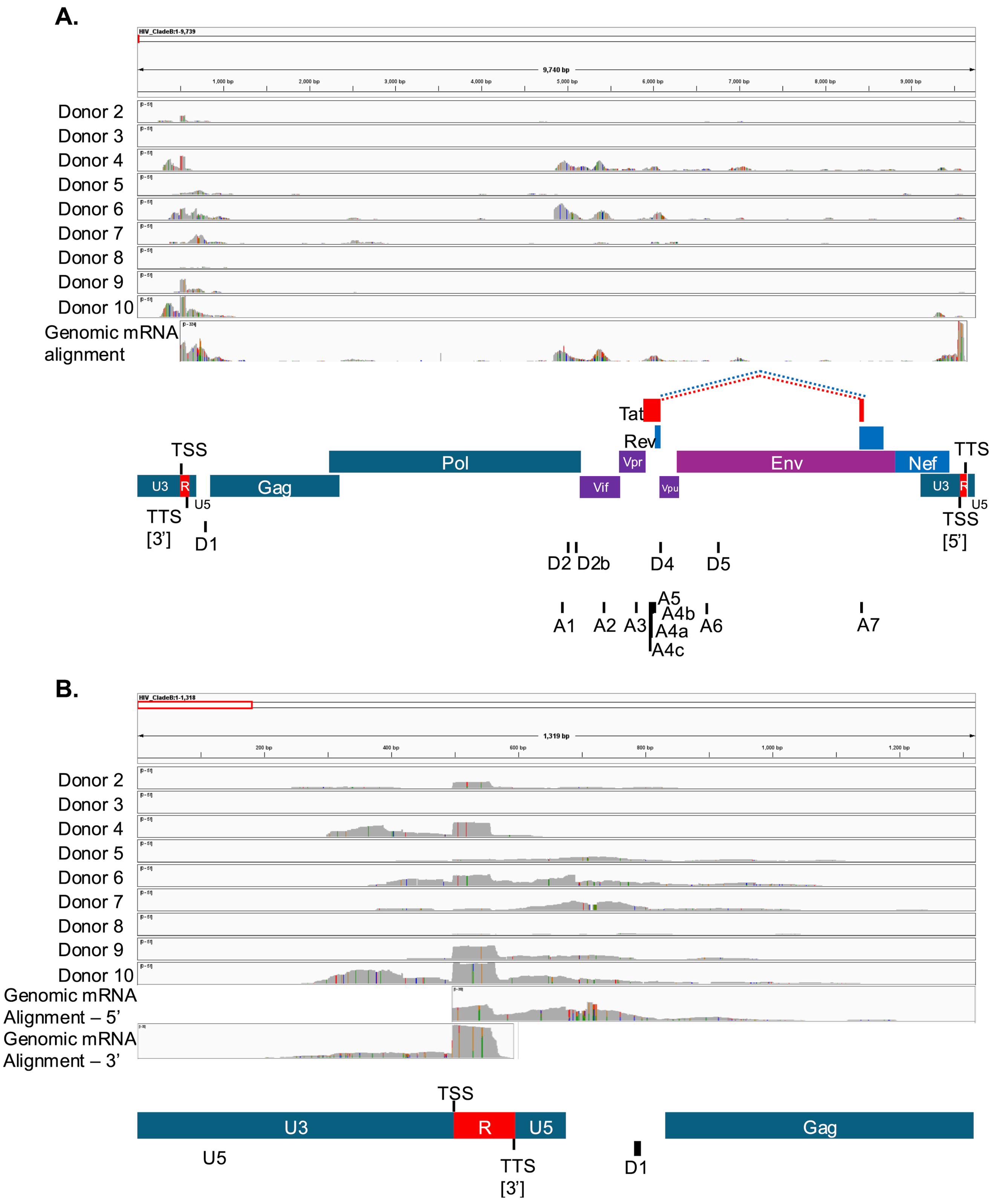
A.
HIV_CladeB:1-9,739
9,740 bp
1,000 bp
2,000 bp
3,000 bp
4,000 bp
5,000 bp
6,000 bp
7,000 bp
8,000 bp
9,000 bp
Donor 2
Donor 3
Donor 4
Donor 5
Donor 6
Donor 7
Donor 8
Donor 9
Donor 10
Genomic mRNA alignment
Tat
Rev
TSS
Pol
Vpr
Env
Nef
TTS
U3
R
Gag
Vif
Vpu
U3
R
U5
TTS [3']
U5
D1
TSS [5']
D2 D2b
D4
D5
A1
A2
A3
A5
A4b
A4a
A4c
A6
A7
B.
HIV_CladeB:1-1,318
1,319 bp
200 bp
400 bp
600 bp
800 bp
1,000 bp
1,200 bp
Donor 2
Donor 3
Donor 4
Donor 5
Donor 6
Donor 7
Donor 8
Donor 9
Donor 10
Genomic mRNA Alignment – 5'
Genomic mRNA Alignment – 3'
TSS
U3
R
U5
Gag
U5
TTS [3']
D1

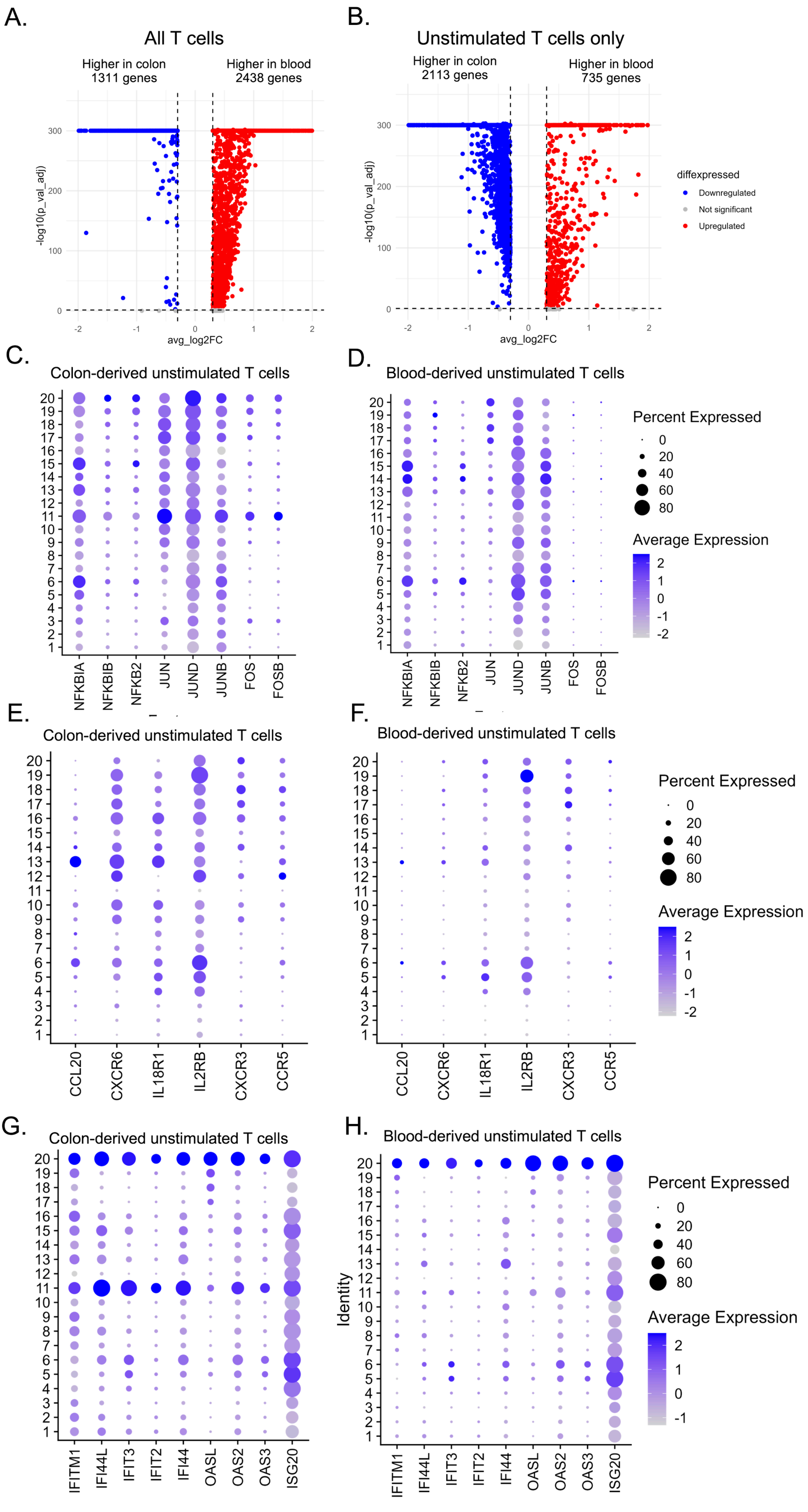
A.
All T cells
Higher in colon
1311 genes
Higher in blood
2438 genes
-log10(p_val_adj)
avg_log2FC
B.
Unstimulated T cells only
Higher in colon
2113 genes
Higher in blood
735 genes
diffexpressed
Downregulated
Not significant
Upregulated
C.
Colon-derived unstimulated T cells
NFKBIA
NFKBIB
NFKB2
JUN
JUND
JUNB
FOS
FOSB
D.
Blood-derived unstimulated T cells
Percent Expressed
Average Expression
E.
Colon-derived unstimulated T cells
CCL20
CXCR6
IL18R1
IL2RB
CXCR3
CCR5
F.
Blood-derived unstimulated T cells
G.
Colon-derived unstimulated T cells
IFITM1
IFI44L
IFIT3
IFIT2
IFI44
OASL
OAS2
OAS3
ISG20
H.
Blood-derived unstimulated T cells
Identity

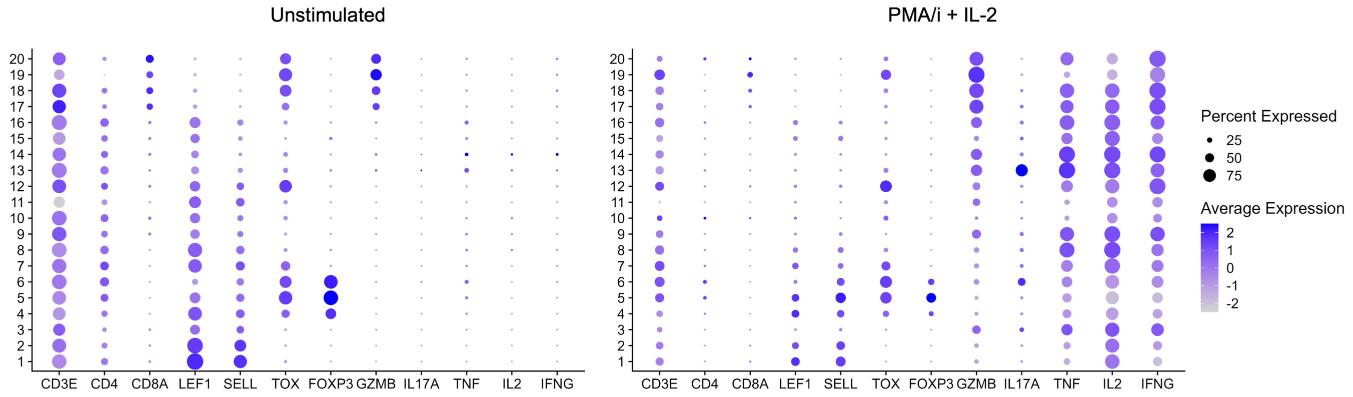

Unstimulated
PMA/i + IL-2
20
19
18
17
16
15
14
13
12
11
10
9
8
7
6
5
4
3
2
1
CD3E
CD4
CD8A
LEF1
SELL
TOX
FOXP3
GZMB
IL17A
TNF
IL2
IFNG
Percent Expressed
25
50
75
Average Expression
2
1
0
-1
-2

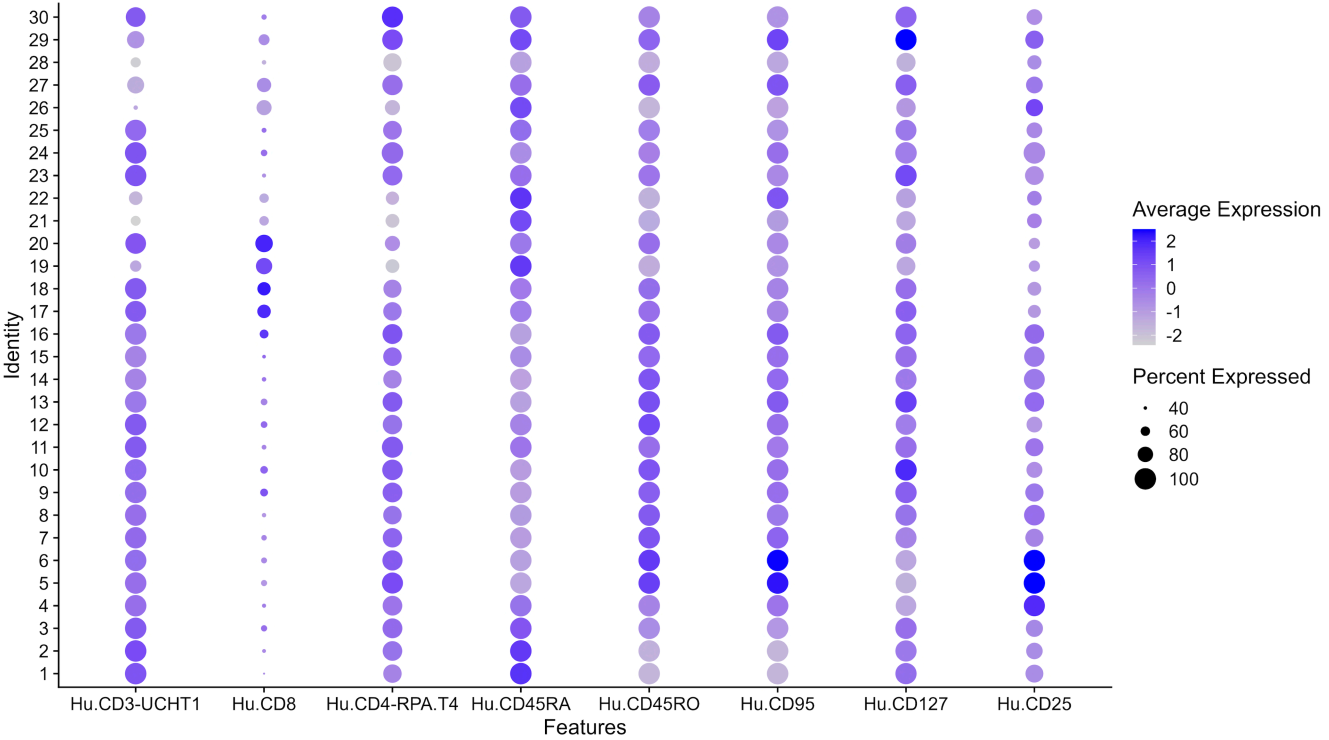

Average Expression
2
1
0
-1
-2
Percent Expressed
40
60
80
100
Identity
30
29
28
27
26
25
24
23
22
21
20
19
18
17
16
15
14
13
12
11
10
9
8
7
6
5
4
3
2
1
Hu.CD3-UCHT1
Hu.CD8
Hu.CD4-RPA.T4
Hu.CD45RA
Hu.CD45RO
Hu.CD95
Hu.CD127
Hu.CD25
Features

A.

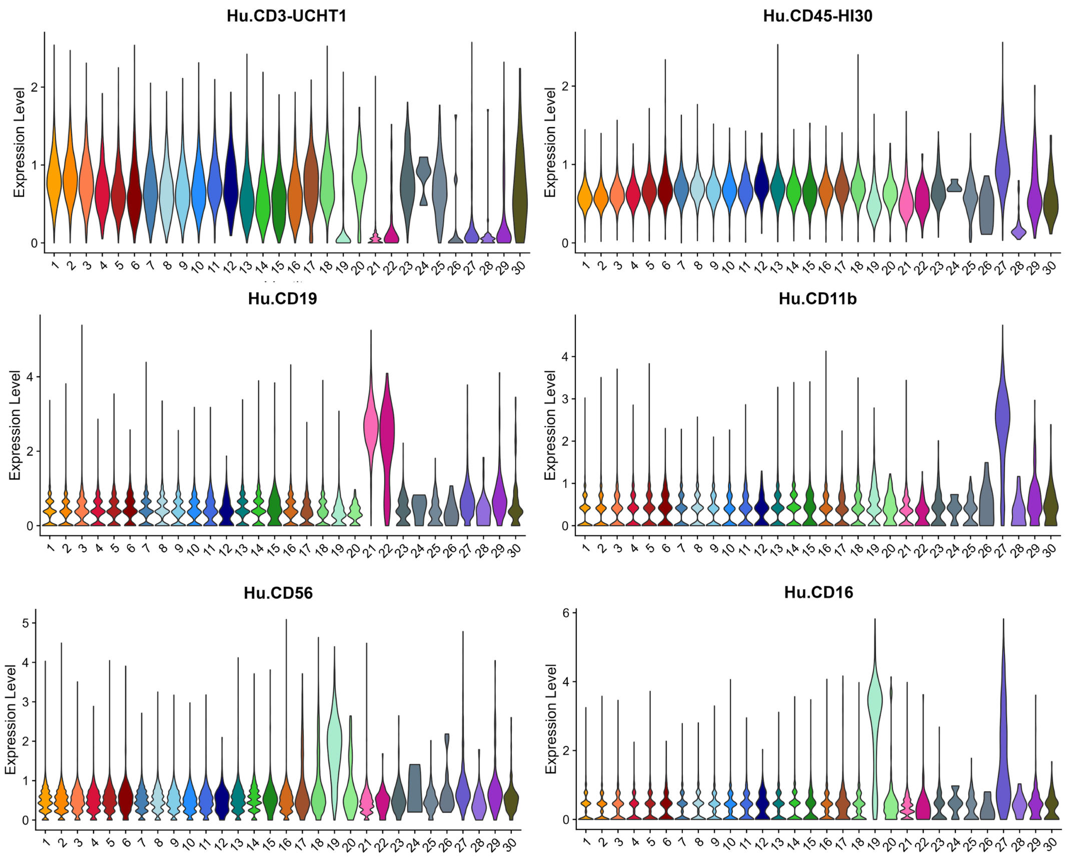
Hu.CD3-UCHT1
Hu.CD45-HI30
Hu.CD19
Hu.CD11b
Hu.CD56
Hu.CD16
Expression Level

B.

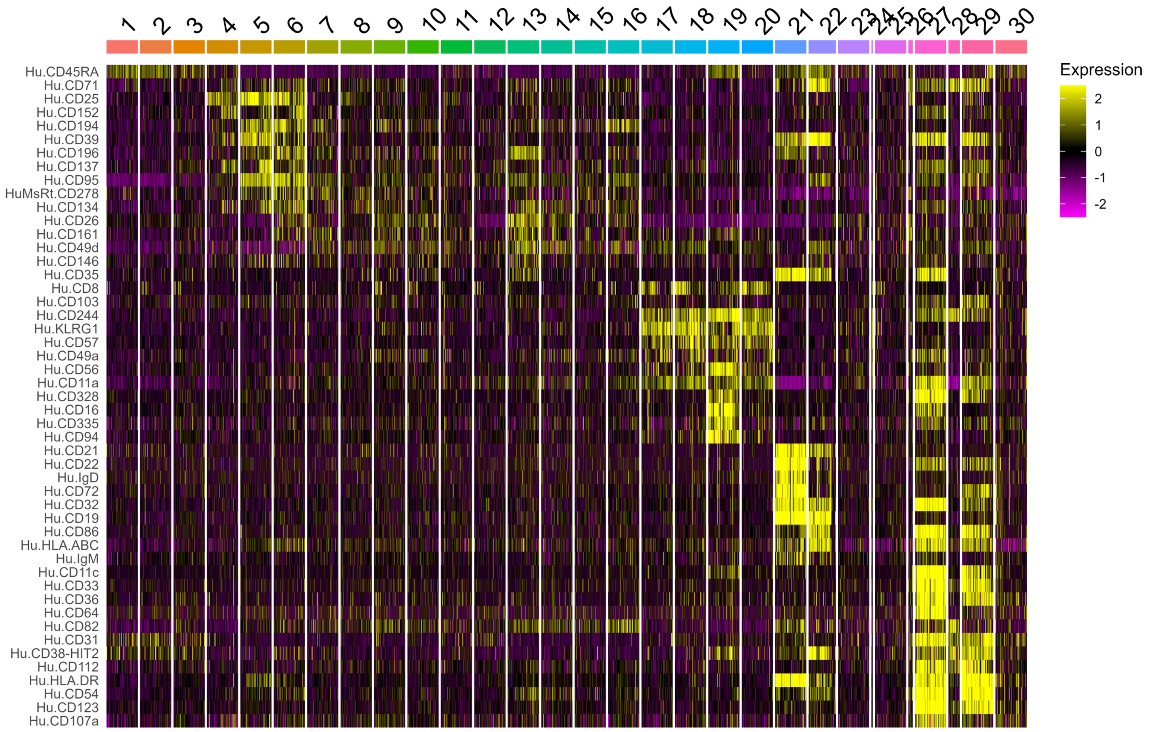
Expression
Hu.CD45RA
Hu.CD71
Hu.CD25
Hu.CD152
Hu.CD194
Hu.CD39
Hu.CD196
Hu.CD137
Hu.CD95
HuMsRt.CD278
Hu.CD134
Hu.CD26
Hu.CD161
Hu.CD49d
Hu.CD146
Hu.CD35
Hu.CD8
Hu.CD103
Hu.CD244
Hu.KLRG1
Hu.CD57
Hu.CD49a
Hu.CD56
Hu.CD11a
Hu.CD328
Hu.CD16
Hu.CD335
Hu.CD94
Hu.CD21
Hu.CD22
Hu.IgD
Hu.CD72
Hu.CD32
Hu.CD19
Hu.CD86
Hu.HLA.ABC
Hu.IgM
Hu.CD11c
Hu.CD33
Hu.CD36
Hu.CD64
Hu.CD82
Hu.CD31
Hu.CD38-HIT2
Hu.CD112
Hu.HLA.DR
Hu.CD54
Hu.CD123
Hu.CD107a

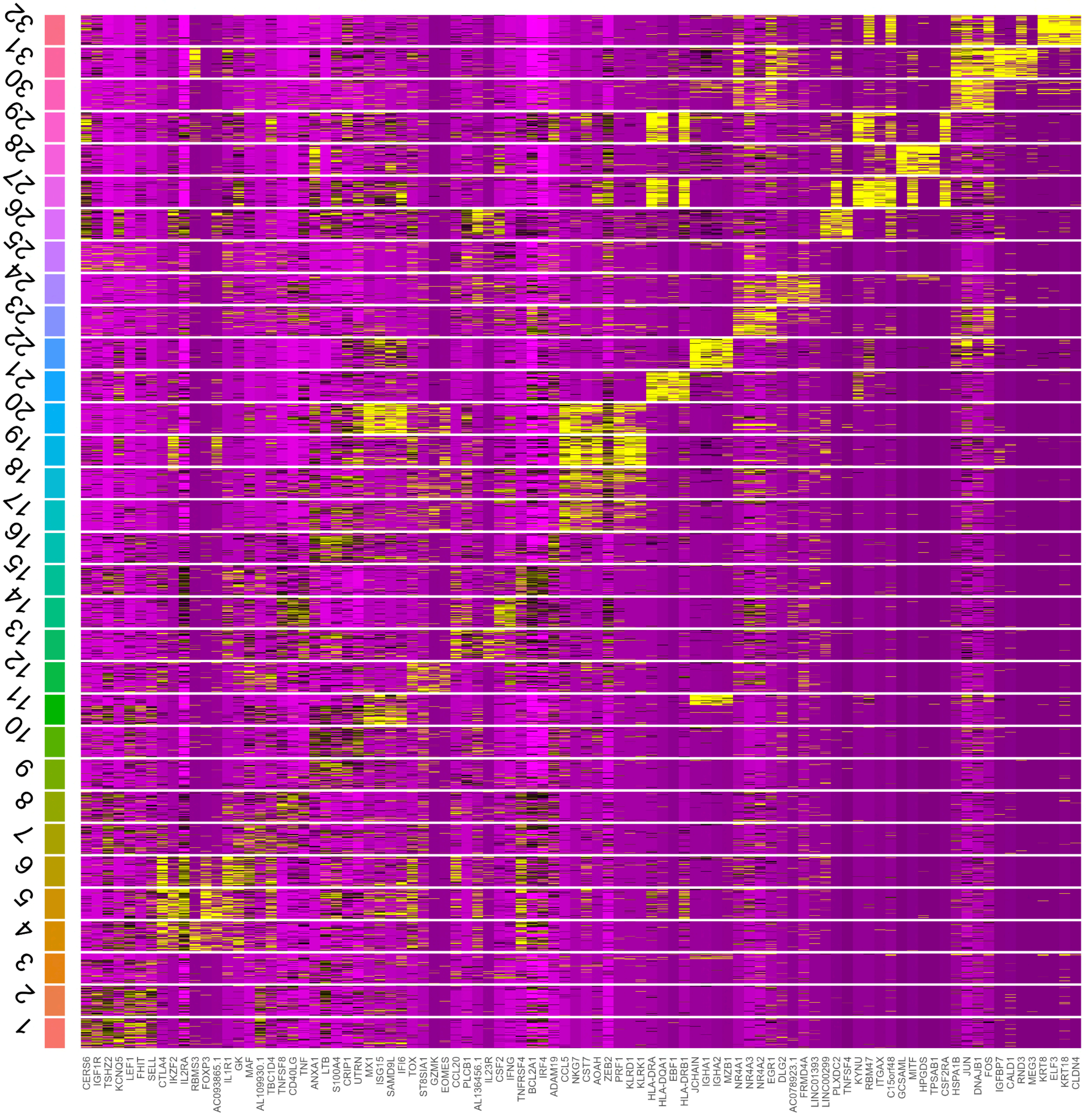
1
2
3
4
5
6
7
8
9
10
11
12
13
14
15
16
17
18
19
20
21
22
23
24
25
26
27
28
29
30
31
32
CERS6
IGF1R
TSHZ2
KCNQ5
LEF1
FHIT
SELL
CTLA4
IKZF2
IL2RA
RBMS3
FOXP3
AC093865.1
IL1R1
GK
MAF
AL109930.1
TBC1D4
TNFSF8
CD40LG
TNF
ANXA1
LTB
S100A4
CRIP1
UTRN
MX1
ISG15
SAMD9L
IFI6
TOX
ST8SIA1
GZMK
EOMES
CCL20
PLCB1
AL136456.1
IL23R
CSF2
IFNG
TNFRSF4
BCL2A1
IRF4
ADAM19
CCL5
NKG7
CST7
AOAH
ZEB2
PRF1
KLRD1
KLRK1
HLA-DRA
HLA-DQA1
EBF1
HLA-DRB1
JCHAIN
IGHA1
IGHA2
MZB1
NR4A1
NR4A3
NR4A2
EGR1
DLG2
AC078923.1
FRMD4A
LINC01393
LINC00299
PLXDC2
TNFSF4
KYNU
RBM47
ITGAX
C15orf48
GCSAML
MITF
HPGDS
TPSAB1
CSF2RA
HSPA1B
JUN
DNAJB1
FOS
IGFBP7
CALD1
RND3
MEG3
KRT8
ELF3
KRT18
CLDN4

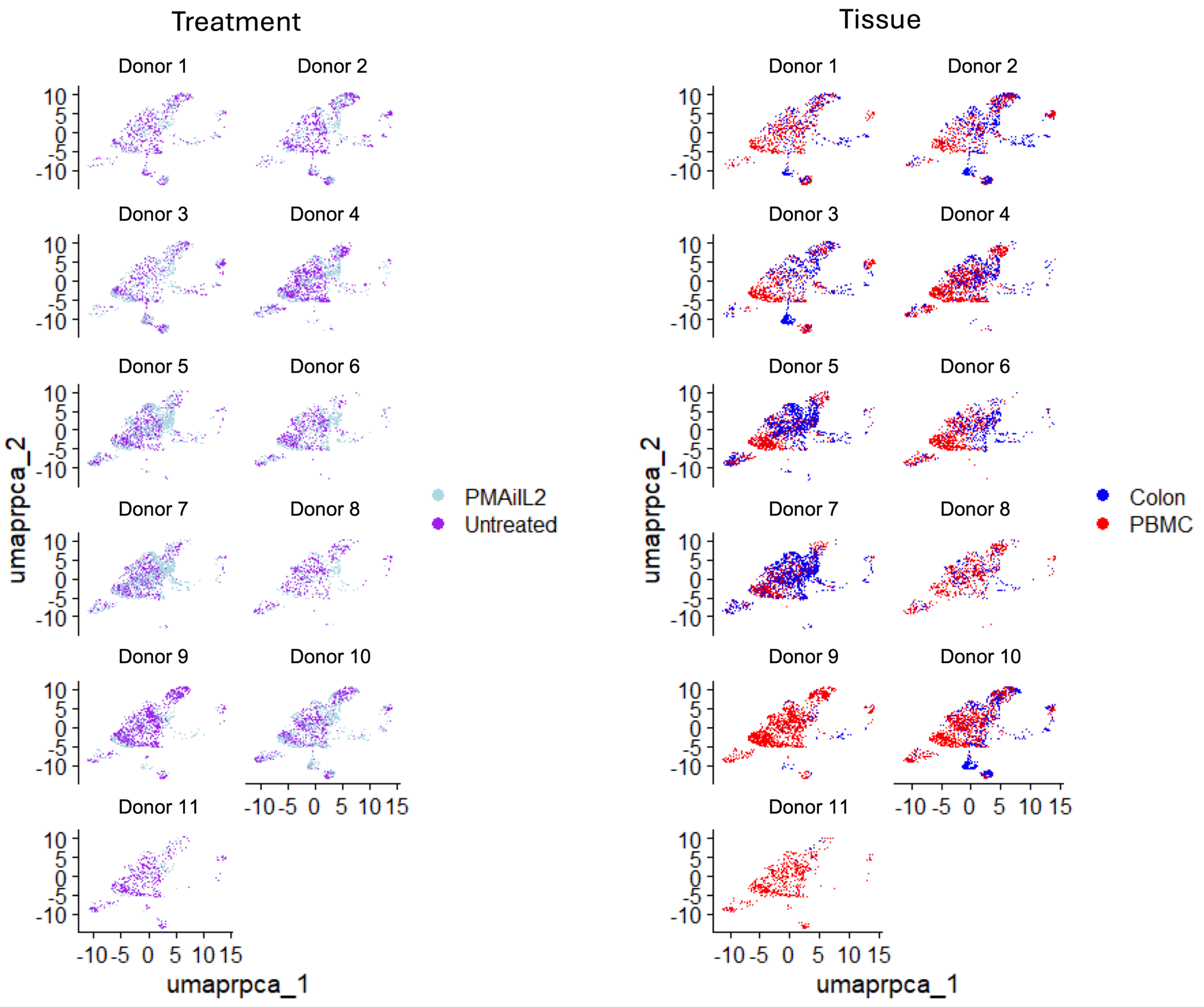

Treatment
Tissue
Donor 1
Donor 2
Donor 3
Donor 4
Donor 5
Donor 6
Donor 7
Donor 8
Donor 9
Donor 10
Donor 11
PMAiIL2
Untreated
Colon
PBMC
umaprpca_1
umaprpca_2

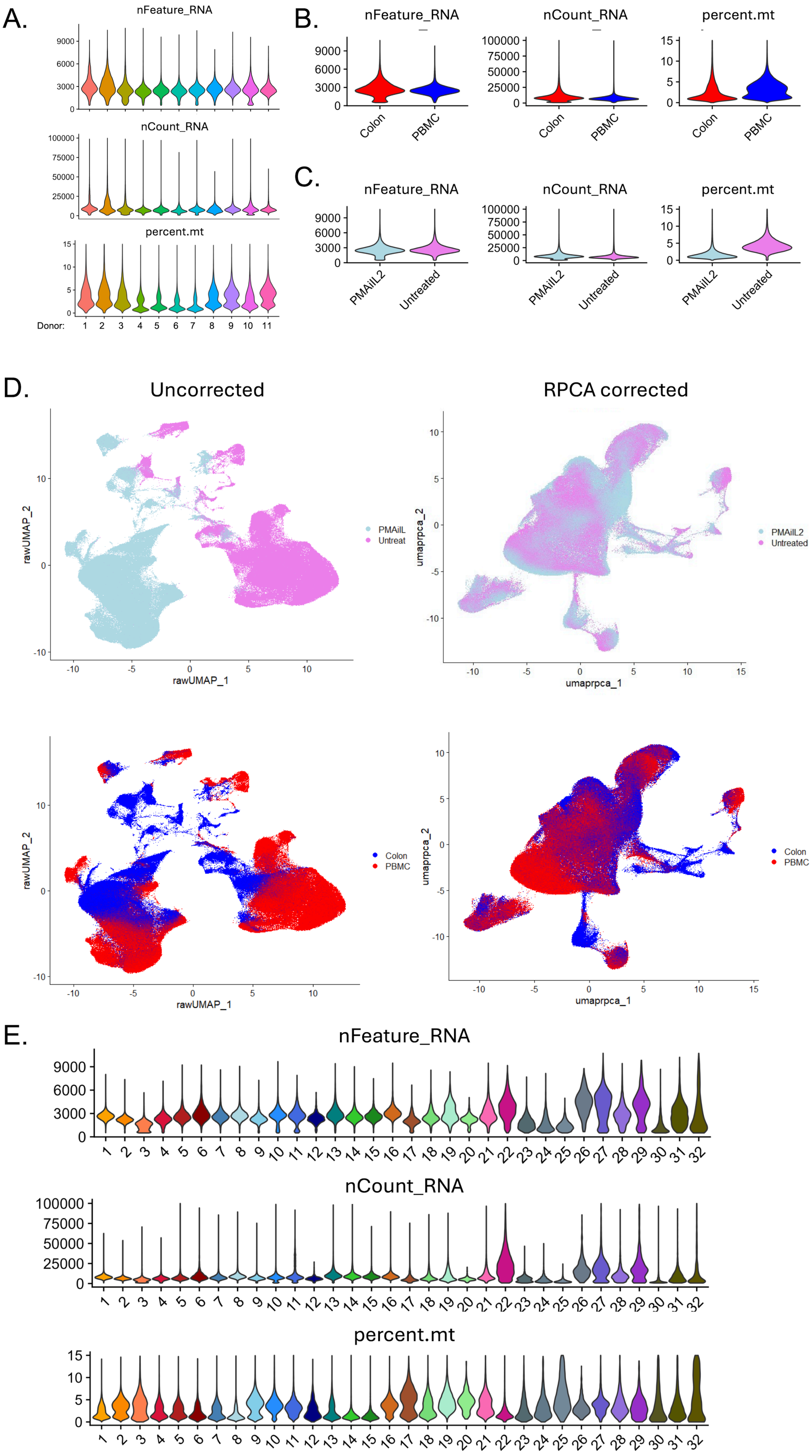

A.
nFeature_RNA
nCount_RNA
percent.mt
Donor:
B.
nFeature_RNA
nCount_RNA
percent.mt
Colon
PBMC
C.
nFeature_RNA
nCount_RNA
percent.mt
PMAiIL2
Untreated
D.
Uncorrected
RPCA corrected
rawUMAP_1
rawUMAP_2
umaprpca_1
umaprpca_2
PMAiIL
Untreat
PMAiIL2
Untreated
Colon
PBMC
E.
nFeature_RNA
nCount_RNA
percent.mt

Supplemental Figure 1: scRNAseq data analysis pipeline.

Data analysis pipeline for this study (see methods). Alignment and single cell expression matrices were created with cellranger (10x Genomics) and parsed in R for initial QC, application of metadata, and merging of a final analysis object. CITEseq samples were additionally processed by demultiplexing of cell hashtag barcodes and application of sample metadata.

Supplemental Figure 2: scRNA-seq data quality and RPCA correction.

A. Violin plots displaying data quality metrics from all analyzed cells from each donor. Top: nFeature_RNA, a metric for the number of detected genes in each cell. Middle: nCount_RNA, a metric for the number of unique molecular identifiers (UMIs) detected within each cell. Bottom: percent.mt, a metric for the number of mitochondrial aligned reads detected within each cell. **B,C**. Violin plots displaying nFeature_RNA, nCount_RNA, and percent.mt detected within each cell, stratified by whether cells were isolated from the colon or blood (**B**) or from PMAi/IL-2 stimulated or unstimulated conditions (**C**). **D**. Uncorrected (left) and RPCA corrected (right) UMAP plots with cells colored by PMAi/IL-2 (light blue) or untreated (purple) stimulation condition, colon (blue) or blood (red). **E**. Violin plots displaying nFeature_RNA, nCount_RNA, and percent.mt detected within each cluster.

Supplemental Figure 3: Visualization of scRNAseq data by donor.

RPCA corrected UMAP visualization of the scRNAseq dataset, separated by individual donor. Left: PMAi/IL-2 treated (light blue) and untreated (purple) cells for each donor. Right: Cells from colon (blue) and blood (red) for each donor.

Supplemental Figure 4: Heatmap of differentially expressed genes from each cluster.

Heatmap of the top 4 (or fewer) differentially expressed genes derived from the top 2000 variable features and calculated from each cluster (Seurat, FindAllMarkers) relative to all other cells. Data from each cluster were downsampled to 100 cells for clear visualization.

Supplemental Figure 5: Surface protein profiles of transcriptome clusters.

Violin plots (**A**) and heat map (**B**) displaying surface antibody staining signal of major immune cell lineage surface markers assessed in cells from each transcriptomic cluster. Data is derived from blood samples from three donors that were analyzed for parallel surface antibody staining and scRNAseq. Not shown are clusters 31 and 32 which were specific to cells derived from the colon.

Supplemental Figure 6: CITEseq surface profiling of T cell lineage markers.

Dot plot displaying surface antibody staining for key T cell lineage markers within each cell cluster. Data is from blood samples from three donors.

Supplemental Figure 7: Activation of cytokine expression by PMAi/IL-2.

Dot plots in all T cell clusters (y-axis) of RNA expression of key T cell markers and cytokines (x-axis) at baseline (left, unstimulated) and after stimulation with PMAi/IL-2 (right).

Supplemental Figure 8: Differential transcript abundance between blood and colon CD4 T cells.

A, **B**. Volcano plots displaying differentially expressed genes (Seurat, FindMarkers, log_2foldchange>0.3, $Pval_{adj}$<0.05 Wilcoxon Rank Sum test) between all (left) or unstimulated (right) blood-derived T cells and colon T cells. Genes with higher expression in colon cells shown in blue,

genes with higher expression in blood cells shown in red. Genes returning $Pval_{adj}$=0 were adjusted to 10^{-300} causing the horizontal line at the top of the plot. **C**-**H**. Dot plots displaying expression of factors differentially expressed between colon and blood-derived cells including NF-κB and AP1 factors (**C**,**D**), cytokines and receptors (**E**,**F**), and Interferon stimulated genes (**G**,**H**) in unstimulated colon-derived T cells (**C**,**E**,**G**) and unstimulated blood-derived T cells (**D**,**F**,**H**).

<u>Supplemental Figure 9: Visualization of scRNAseq reads mapping to the proviral genome.</u>

A. Mapping of scRNAseq reads to a consensus clade B HIV proviral genome and to a corresponding viral genomic RNA was visualized using the Integrated Genomics Viewer (IGV). HIV map includes viral splicing donor (D1-D5) and acceptor (A1-A7) sites. Bam files were filtered to only include cell barcodes matching final cell matrix, merged by donor, and visualized in IGV. Coloration in coverage plots correspond to nucleotide variants relative to reference. **B**. IGV coverage plots of 5' end of proviral genome and viral genomic RNA. Peak in coverage near position 500bp corresponds to predicted transcription start in Clade B reference at position 496.

<u>Supplemental Figure 10: Frequency and expression level of vRNA+ cells across clusters.</u>

A. The frequency of vRNA+ cells per million cells across all transcriptomic clusters, including blood and colon is shown. **B.** HIV expression level for all vRNA+ cells across the transcriptomic clusters is shown. Each dot represents a single infected cell.

Supplemental Table 1: Study participants

Supplemental Table 2: Sample list

Supplemental Table 3: Cluster annotation

Supplemental Table 4: DEGs between blood and colon CD4 T cells

Supplemental Table 5: MSigDB_Hallmark_Top 500 upregulated DEGs in blood

Supplemental Table 6: MSigDB_Hallmark_Top 500 upregulated DEGs in colon

Supplemental Table 7: DEGs between unstimulated blood and colon CD4 T cells

Supplemental Table 8: Abundance and frequency of vRNA+ cells across transcriptomic clusters

Supplemental Table 9: HIV aligned read data annotated with sample details

Supplemental Table 10: HIV DEGs – all samples

Supplemental Table 11: HIV DEGs - unstimulated blood cells

Supplemental Table 12: HIV DEGs - unstimulated colon cells

Supplemental Table 13: HIV DEGs - stimulated blood cells

Supplemental Table 14: HIV DEGs - stimulated colon cells

Supplemental Table 16: MSigDB_Hallmark_2020_HIV DEGs All samples

Supplemental Table 17: KEGG_2021_HIV DEGs all samples

Supplemental Table 18: KEGG_2021_HIV DEGs stimulated blood T cells

Supplemental Table 15: Differentially expressed surface proteins in vRNA+ cells
